# Design and Bench Evaluation of a Sensor-Integrated Prosthetic-Leg Prototype

**DOI:** 10.3390/s26196341

**Published:** 2026-10-08

**Authors:** Turar Seitkassymov, Yerkebulan Nurgizat, Nursultan Zhetenbayev, Aidos Sultan, Gani Sergazin, Kassymbek Ozhikenov

**Affiliations:** 1 Department of Robotics and Technical Means of Automation, Satbayev University, Almaty 050013, Kazakhstann.zhetenbaev@aues.kz (N.Z.);; 2 Department of Aerospace and Electronic Engineering, Institute of Telecommunications and Automation, Almaty University of Power Engineering and Telecommunications, Almaty 050013, Kazakhstan; 3 Department of Science and Innovations, Mukhametzhan Tynyshbayev ALT University, Almaty 050013, Kazakhstan

**Keywords:** prosthetic-leg prototype, surface electromyography, inertial measurement unit, sensor-triggered position command, multi-material foot, additive manufacturing, finite-element analysis, bench testing

## Abstract

This study presents the design and preliminary bench evaluation of a sensor-integrated prosthetic-leg prototype. The platform combines a multi-material PAHT-CF/TPU 95A foot, articulated knee and ankle mechanisms, two surface electromyography channels, an MPU6050 inertial measurement unit, an Arduino Uno controller, and MG995 servomotors. EMG threshold crossings select discrete knee–servomotor commands, while an accelerometer-derived inclination estimate selects between two ankle–servomotor commands. The evaluation comprised exploratory finite-element analyses, separate static compression tests, and qualitative demonstrations of the sensor-to-actuator pathways. The foot model predicted a maximum displacement of 25.96 mm under 400 N, whereas a separate compression test recorded approximately 7 mm at 1000 N; these values do not constitute quantitative model validation because the loading, constraints, and displacement measures differed. The shank model produced a maximum equivalent stress of 23.89 MPa, and a separate shank–knee assembly test reached approximately 1.85 kN. The foot–ankle connection produced a stress estimate of 203.6 MPa against an assumed strength value of 60 MPa, identifying a critical design limitation. Functional observations demonstrated sensor-triggered command execution, but joint tracking accuracy, response time, actuator output under load, and control repeatability were not quantified. The prototype therefore provides an initial laboratory platform for investigating mechanical and sensing integration; structural redesign and quantitative bench testing are required before any load-bearing or clinical use can be considered.

## 1. Introduction

Loss of a lower limb represents a major medical, social, and engineering challenge because it directly affects an individual’s mobility, independence, work capacity, and overall quality of life. Following amputation, patients face not only restrictions in physical activity but also the need for prolonged rehabilitation, adaptation to a prosthetic device, and restoration of a stable gait pattern. Globally, the demand for rehabilitation continues to increase. According to the World Health Organization, approximately 2.4 billion people worldwide live with health conditions that may benefit from rehabilitation services, and this number is expected to rise further as a result of population aging and the increasing prevalence of chronic diseases [1].

Prostheses and orthoses, which are classified as assistive technologies, play a particularly important role within rehabilitation systems. According to the joint global report by the WHO and UNICEF, more than 2.5 billion people worldwide require at least one assistive product; however, nearly 1 billion people lack access to such technologies. In low- and middle-income countries, access to assistive products may cover only approximately 3% of the actual need [2]. The accessibility problem is particularly critical in prosthetic and orthotic care. The WHO reports that only approximately one in ten individuals in need has access to assistive products, including prostheses and orthoses, primarily because of high costs, shortages of qualified specialists, insufficient funding, and limited healthcare infrastructure [3].

The growing number of patients requiring prosthetic rehabilitation is associated with both chronic diseases and traumatic injuries. A study of the global prevalence of traumatic amputations based on Global Burden of Disease data reported that, in 2017, approximately 57.7 million people worldwide were living with limb amputation resulting from traumatic causes. The leading causes included falls, road traffic accidents, other transport-related injuries, and mechanical trauma [4]. These findings demonstrate that limb amputation is not only a medical issue but also a substantial socioeconomic challenge, as restoration of mobility requires prosthetic solutions that are accessible, functional, and adaptable to individual users.

Modern lower-limb prostheses have undergone substantial development, progressing from passive mechanical devices to microprocessor-controlled and actively powered systems. Passive prostheses are characterized by mechanical simplicity and relatively low cost; however, their functionality is inherently limited because they cannot actively adapt to the user’s movement intention, gait phase, or changing locomotion conditions. Microprocessor-controlled and active prostheses provide a higher level of functionality, but their widespread adoption remains constrained by high cost, maintenance complexity, and dependence on specialized components. Consequently, the development of low-cost, sensor-integrated prosthetic systems capable of providing basic adaptive motion while maintaining structural simplicity and manufacturing accessibility remains an important challenge in contemporary biomedical engineering.

One promising approach involves the use of biosignals and inertial measurements for prosthetic motion control. Electromyographic signals provide information on the user’s muscle activity and can therefore be employed to infer movement intention. In a systematic review, Ahkami et al. reported that interest in the application of EMG signals for lower-limb prosthesis control has increased substantially over the past decade; however, commercially available lower-limb prostheses still rarely employ EMG as the primary control input [5]. This indicates the existence of a technological gap between research developments and practically accessible prosthetic solutions.

Inertial measurement units provide an additional source of information regarding human movement and typically integrate accelerometers and gyroscopes. IMU sensors can be used to determine the orientation and dynamic behavior of limb segments, which is particularly important for gait-phase estimation, foot-angle assessment, and motion stabilization. Xu and Wang demonstrated the feasibility of using two IMU modules to recognize locomotion modes in users of robotic lower-limb prostheses, including level-ground walking, stair ascent and descent, and locomotion on inclined surfaces [6]. Therefore, the integration of muscle-activity information with inertial measurements may increase the amount of relevant information available to the control system and provide more robust interaction between the user and an active prosthetic device.

Another important research direction concerns improvements in the mechanical design of prostheses, particularly the prosthetic foot module. The foot absorbs impact loads during initial contact with the supporting surface, contributes to the rollover phase, and generates propulsion during walking. Excessive structural stiffness may adversely affect locomotion biomechanics, whereas insufficient stiffness may reduce stability and the efficiency of force transmission. Consequently, recent research has increasingly focused on the development of prosthetic feet incorporating flexible forefoot regions and controllable or variable stiffness. For example, Um et al. proposed a 3D-printed prosthetic foot with nonlinear toe-joint stiffness, in which geometric configuration, the bending region, and an auxetic structure were employed to approximate the mechanical behavior of the prosthesis to the natural biomechanics of the human foot [7].

Integrated sensing and actuation are established features of powered lower-limb prostheses, including the open-source knee–ankle platform reported in [8]. Accordingly, the combination of commercially available EMG sensors, an IMU, a microcontroller, and servomotors does not by itself establish a new sensing or control principle. The present study instead investigates a specific prototype configuration in which a multi-material foot with passive forefoot compliance is mechanically integrated with separately commanded knee and ankle mechanisms.

Our previous study [9] focused on an EMG-controlled semi-active knee architecture and already included inertial sensing. The present work extends the hardware scope to a PAHT-CF/TPU 95A foot and an articulated ankle, with two EMG inputs assigned to discrete knee commands and an accelerometer-derived inclination input assigned to ankle commands. The contribution is therefore an extension of the mechanical configuration and its bench-level investigation. Improved intent recognition, superior controller performance, and improved walking function relative to [9] have not been demonstrated.

The objective of the present study is to document this integrated prototype configuration, examine selected structural components through exploratory finite-element analysis and separate compression tests, and demonstrate the generation of discrete actuator commands from the sensor inputs. Particular attention is given to the limitations of the current design, including the foot–ankle connection, the absence of measured prosthetic-joint position feedback, and the restricted validation depth of the sensor pathways. The work is therefore positioned as a design and bench-evaluation study rather than validation of a clinically usable prosthesis. A direct comparison of the present study scope with related prosthetic platforms is provided in Table 1.

## 2. Materials and Methods

### 2.1. Analysis of Contemporary Approaches to the Design of Active Lower-Limb Prostheses

At the initial stage of the study, contemporary approaches to the design of active lower-limb prostheses were analyzed. Unlike an isolated prosthetic foot module, a complete lower-limb prosthesis represents an integrated biomechanical and mechatronic system comprising a load-bearing structure, prosthetic foot, ankle and knee joints, actuators, sensing system, power supply, and control unit. Accordingly, the design of such a prosthesis must consider not only the strength and mass of individual components but also the coordinated operation of the joint mechanisms, load transmission, user stability, locomotion energy efficiency, and the ability of the system to adapt to the user’s movement intention.

Modern lower-limb prostheses can generally be classified into four principal categories: passive mechanical prostheses, microprocessor-controlled prostheses, semi-active systems, and actively powered robotic prostheses. Passive prostheses are characterized by structural simplicity, relatively low cost, and high reliability; however, their functionality remains limited. Such systems are unable to actively generate additional joint torque, adapt to different phases of the gait cycle, or modify their movement parameters according to the user’s locomotor intention. Their primary functions are therefore limited to body-weight support, passive articulation, and mechanical stabilization.

Microprocessor-controlled knee modules represented an important stage in the evolution of lower-limb prosthetic technology. These systems employ joint-angle, angular-velocity, and load sensors in combination with microcontroller-based algorithms to regulate resistance within the knee joint according to the current phase of movement. Microprocessor-controlled knee systems have been shown to improve walking safety and energy efficiency in individuals with transfemoral amputation [10]. Nevertheless, such systems generally do not generate substantial positive mechanical work; instead, they primarily regulate damping or resistance to joint motion. Furthermore, their high cost, the complexity of individual adjustment, and the requirement for specialized servicing limit their widespread accessibility.

Semi-active prostheses represent another important development direction. In such systems, actuators do not necessarily produce fully powered joint motion but instead modify the mechanical characteristics of the prosthetic joint. Examples include systems based on variable damping, magnetorheological brakes, controllable shock absorbers, or low-power servomotors. Their principal advantage is lower energy consumption compared with fully powered prostheses. However, semi-active systems have a limited capacity to generate positive mechanical work, which is particularly important during stair ascent, inclined-surface locomotion, and active push-off.

Actively powered robotic lower-limb prostheses are intended to reproduce not only passive weight-bearing functions but also active motion at the knee and/or ankle joints. An active prosthesis should therefore be considered a mechatronic system in which actuator selection, mechanical design, and control-algorithm development constitute the key engineering challenges [11]. In such systems, electric motors, transmission mechanisms, elastic elements, and sensors must operate in a coordinated manner to produce movement patterns approaching the natural biomechanics of human locomotion. Nevertheless, the main limitations of active prostheses remain their mass, power consumption, cost, safety requirements, and control complexity.

One well-established direction in this field is the development of powered knee–ankle prostheses, which integrate active actuation at both the knee and ankle joints. Sup et al. presented a self-contained powered knee–ankle prosthesis intended for individuals with transfemoral amputation [12]. Other studies have investigated powered knee prostheses incorporating antagonistic actuator configurations to improve gait symmetry [13]. Similarly, active ankle prostheses have demonstrated the potential to improve the energetic characteristics of walking by generating mechanical power during the push-off phase [14]. Collectively, these studies indicate that actively powered joints can substantially improve prosthetic functionality, although they require more sophisticated mechatronic architectures.

In recent years, increasing attention has also been directed toward open-source and modular platforms for active prostheses. In [8], an open-source bionic leg integrating powered knee and ankle modules, a sensing system, and a unified control architecture was presented. The importance of such systems lies not only in their functional capabilities but also in their reproducibility, modifiability, and applicability in research laboratories. Nevertheless, these platforms remain technically complex and require precise actuator design, careful controller tuning, and extensive experimental validation.

The development of control strategies constitutes another critical area of research in active lower-limb prosthetics. Commonly investigated approaches include finite-state machines, impedance control, gait-phase-based control, user-intent recognition, and biosignal-based control [15]. Finite-state-machine strategies enable switching between predefined operating states corresponding to different gait phases; however, their performance depends strongly on reliable detection of transition events. Impedance control enables regulation of the mechanical relationship between joint motion and external loading but generally requires individual parameter tuning for each user. Phase-variable control treats gait as a continuous process and may provide smoother transitions between movement states; however, its effectiveness depends on reliable estimation of the current locomotion phase.

To enhance the adaptability of active prostheses, modern systems employ multimodal sensing architectures that may include encoders, force or load sensors, inertial measurement units, electromyographic sensors, and pressure sensors. Real-time multiclass approaches have been developed to recognize user intent in powered lower-limb prostheses [16]. The use of EMG information combined with data from previous gait cycles has also been shown to improve the control of powered prosthetic legs across different locomotion modes [17]. Furthermore, the fusion of EMG signals with mechanical sensor data can improve the accuracy and robustness of user-intent recognition [18]. These findings support the integration of biosignals and mechanical measurements within a unified control framework.

Inertial measurement units are also widely employed in active and robotic prosthetic systems. IMUs enable estimation of limb-segment orientation, inclination angle, acceleration, and movement dynamics. Whereas EMG signals primarily reflect the user’s neuromuscular activity and intended movement, IMU data characterize the actual motion and spatial orientation of the prosthetic segments. Accordingly, combining EMG and IMU sensing is particularly suitable for a sensor-integrated control system: EMG signals may be used to estimate user intention, whereas IMU measurements can provide feedback regarding foot orientation, shank position, and gait phase.

Another important issue is the provision of feedback to the prosthesis user. Most conventional prostheses do not provide natural sensory information regarding ground contact, applied load, or joint position. Gait-synchronized vibrotactile feedback has been shown to influence walking parameters in individuals with lower-limb amputation [19]. Although such feedback is not essential for a basic laboratory prototype, these approaches represent an important direction for future development, in which prosthetic systems are designed not only to control movement but also to partially restore the sensory interaction between the user and the artificial limb.

Recent studies further emphasize that an active lower-limb prosthesis should be designed as an integrated system rather than as a collection of independent components. Robotic prosthetic legs have therefore been investigated from the combined perspectives of mechanical design, control, and system implementation, highlighting the necessity of coordinating mechanical structures, actuators, sensors, and algorithms [20]. Phase-based control approaches for powered knee–ankle prostheses have also been proposed to accommodate both rhythmic and non-rhythmic movements [21]. In addition, locomotion-mode recognition accuracy can be improved by introducing an appropriate decision delay during transitions between locomotion modes [22]. These studies demonstrate that the reliability of an active prosthesis depends not only on the quality of its mechanical components but also on effective sensor processing and robust control logic.

The literature reviewed in [8,10,11,12,13,14,15,16,17,18,19,20,21,22] shows that powered and sensor-integrated lower-limb prostheses are already well established as a research field. The relevant gap for the present work is therefore not the existence of EMG, IMU sensing, or powered joints themselves, but the engineering accessibility of a modular platform that can be fabricated and studied with commonly available laboratory resources. This distinction is important because the present prototype does not reproduce the actuator power, sensing density, control sophistication, or clinical validation depth of mature powered systems such as [8,20].

**Figure 1:**
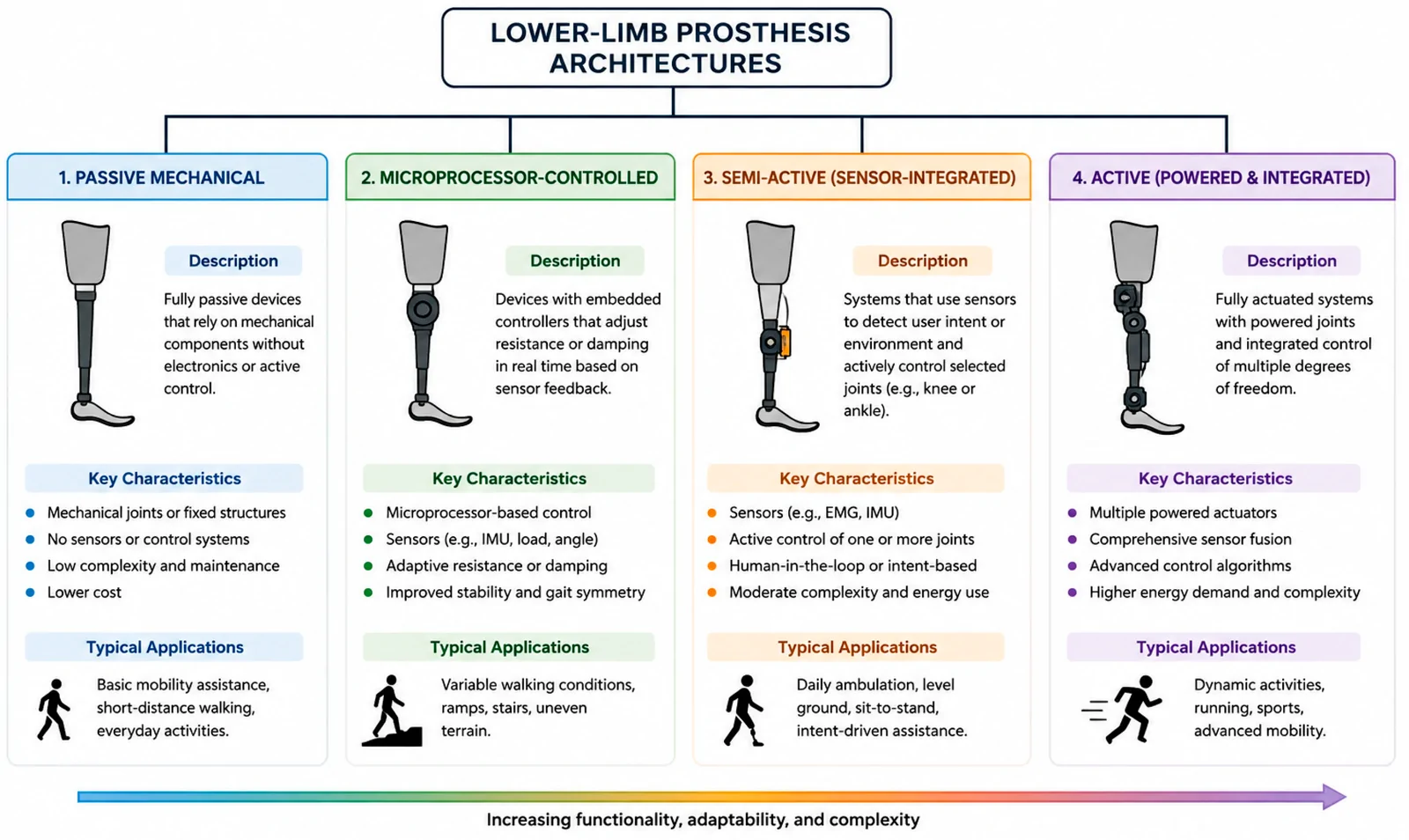
Classification of contemporary lower-limb prosthesis architectures, including passive mechanical, microprocessor-controlled, semi-active, and actively powered sensor-integrated systems, synthesized from the literature [9,14,19,23].

For the present study, the prototype is treated as an accessible sensor-integrated mechatronic research platform (Figure 1). Its articulated knee and ankle mechanisms allow bench demonstration of commanded movements, while the multi-material foot is intended to provide passive forefoot compliance. Shock attenuation, energy return, rollover behavior during gait, user stability, and human load-bearing performance were not measured. The component-loading tests and functional demonstrations therefore address separate engineering questions and do not establish the performance of an assembled load-bearing prosthesis (Table 2).

Accordingly, the Section 2.2 and Section 2.3 focus on the documented configuration and its bench-level evidence rather than on claims of clinical or gait-level functionality. The prototype is used to identify structural limitations, demonstrate sensor-triggered actuation pathways, and define the quantitative measurements required for the next design iteration.

### 2.2. Structural Configuration and Materials of the Developed Prosthesis

**Figure 2:**
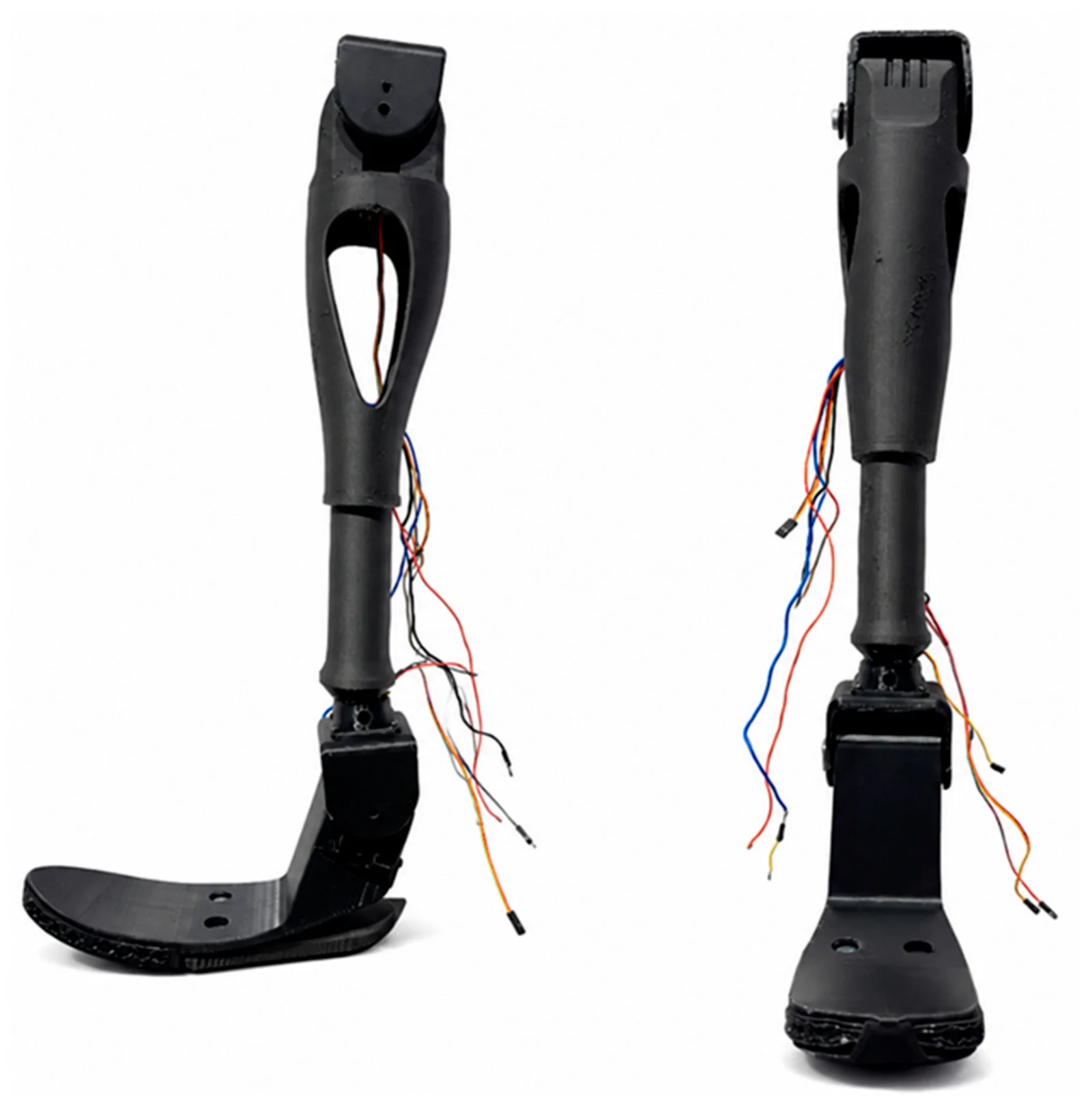
Overall configuration of the prosthetic-leg bench prototype.

A prosthetic-leg bench prototype was developed to examine the integration of an additively manufactured mechanical structure with commercially available sensing and actuation components. The architecture comprises a multi-material foot, an articulated ankle mechanism, a load-bearing shank, an articulated knee mechanism, an upper socket-like interface, servomotors, and a compact electronic control subsystem. The current study evaluates these elements as a laboratory research platform rather than as a clinically fitted prosthesis. The overall prototype configuration is shown in Figure 2.

The mechanical architecture follows a serial arrangement from the upper interface through the knee, shank, ankle, and foot. The articulated joints provide commanded relative motion during bench testing, whereas the compliant forefoot deforms passively. Functions such as shock attenuation, energy return, physiological rollover, and stable user support remain design objectives and were not quantified in the present experiments.

The structural configuration was organized as a serial kinematic chain for defining load transfer and relative joint motion. External loading applied to the upper structure is transmitted through the knee region and shank toward the ankle–foot assembly. This representation is used only to define the mechanical arrangement; it does not establish physiological gait mechanics or safe load transmission through the complete assembled prototype. The main structural components and their roles are summarized in Table 3.

For kinematic description, the knee and ankle were represented as revolute articulations. Let $q_{k}$ denote the relative angle between the proximal link and the shank, and $q_{a}$ denote the relative angle between the shank and the foot. These mechanical coordinates are distinct from the numerical commands sent to the servomotors. Their relationship depends on transmission geometry, mounting offsets, mechanical compliance, and calibration. Consequently, a servomotor command of 90 degrees does not establish a measured knee or ankle joint angle of 90 degrees. The present experiments did not determine command-to-joint mappings or a dynamic model of the assembled leg.

**Figure 3:**
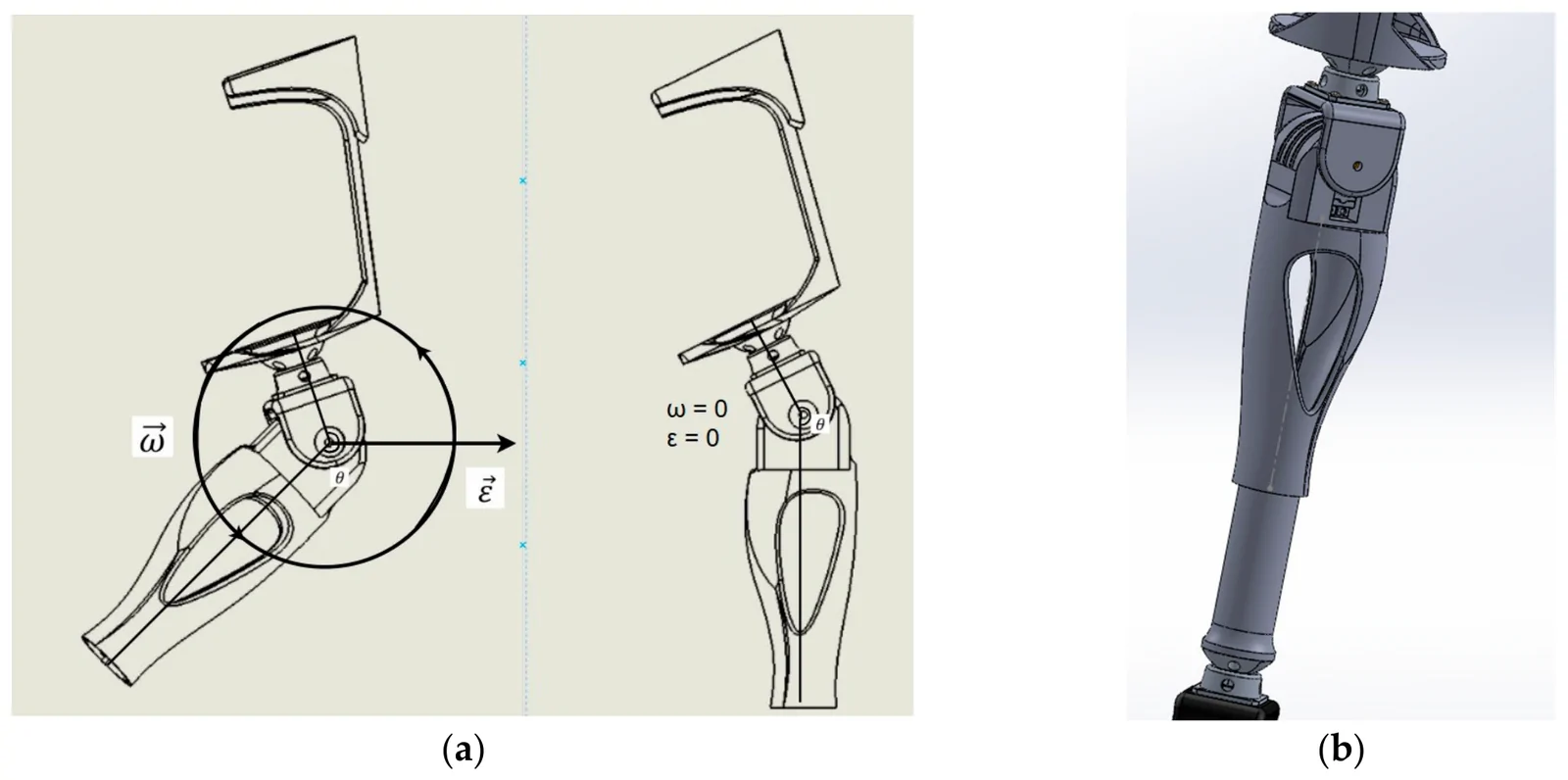
Knee mechanism: (**a**) schematic definition of relative link rotation; (**b**) CAD configuration. In the schematic, ω and ε denote angular velocity and angular acceleration, respectively, and the curved arrow indicates the positive rotational sense. Numerical servomotor commands are distinct from measured prosthetic-joint angles.

The knee mechanism was designed as a single-axis articulated element compatible with servomotor actuation. Its schematic representation identifies the nominal axis of relative rotation and is used to describe the geometry of the bench mechanism; no claim is made that the commanded servomotor positions reproduce physiological knee kinematics under load. The knee mechanism and its nominal rotational coordinate are shown in Figure 3.

**Figure 4:**
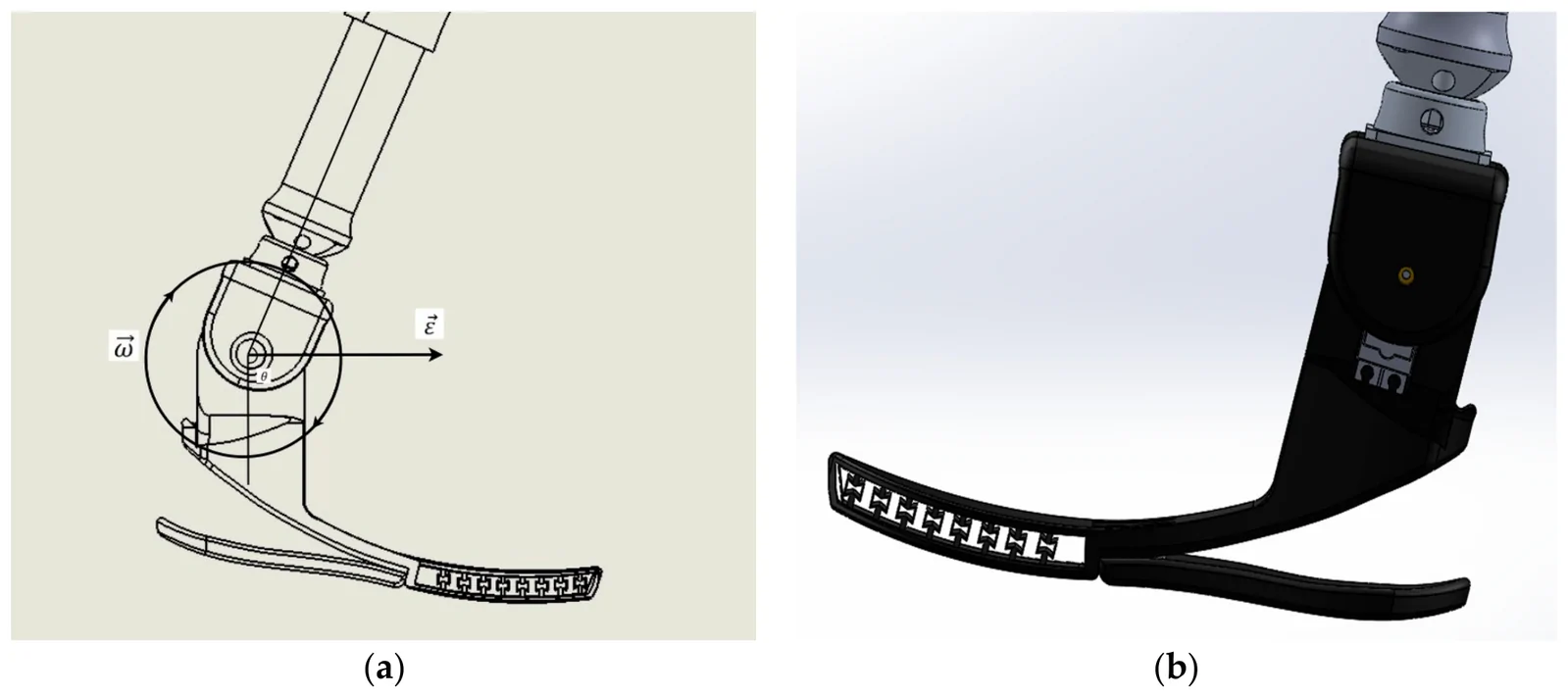
Ankle–foot mechanism: (**a**) schematic definition of foot rotation relative to the shank; (**b**) CAD configuration with a compliant forefoot region. In the schematic, the curved arrow indicates the positive rotational sense of the relative ankle–foot coordinate.

The ankle mechanism was similarly represented as a revolute articulation between the shank and foot. The corresponding mechanical coordinate describes foot rotation relative to the shank, whereas the MPU6050-based control variable described below is a sensor-frame inclination estimate. The two quantities should not be interpreted as equivalent unless independent calibration is performed. The corresponding ankle–foot mechanism is shown in Figure 4.

The foot module combines a relatively rigid PAHT-CF structural region with a TPU 95A forefoot region intended to provide passive compliance. The present study does not quantify energy return, impact attenuation, gait rollover, or the relationship between the deformation profile and physiological foot stiffness; the multi-material arrangement is evaluated only through the reported numerical and separate compression-test observations.

PAHT-CF was selected for regions requiring relatively high stiffness and dimensional stability, whereas TPU 95A was used in the compliant forefoot. The material assignment therefore separates rigid load-bearing geometry from a flexible region, but the effective mechanical properties of the printed parts depend on manufacturing direction, infill, layer bonding, and process settings. The material assignments and functional roles are summarized in Table 4.

The components were fabricated by fused deposition modeling using a Bambu Lab X1 printer (Bambu Lab, Shenzhen, China). PAHT-CF (Bambu Lab, Shenzhen, China) was selected for regions requiring relatively high stiffness and dimensional stability, whereas TPU 95A (Bambu Lab, Shenzhen, China) was used for the compliant forefoot region. The components were fabricated by fused deposition modeling using a Bambu Lab X1 printer (Bambu Lab, Shenzhen, China).

The resulting configuration provides a laboratory platform for evaluating selected structural components and sensor-triggered actuation pathways. It should not be interpreted as a mechanically validated prosthesis for human load-bearing use.

### 2.3. Sensor Architecture, Command Logic, and Bench Evaluation Methods

The controller implements two sensor-triggered command pathways. The EMG inputs select a discrete knee–servomotor command, and an accelerometer-derived inclination estimate selects a discrete ankle–servomotor command. The Arduino controller does not independently measure the knee and ankle joint angles and does not calculate a tracking error between commanded and measured prosthetic-joint positions. Accordingly, the implemented architecture is described as sensor-triggered position-command control rather than closed-loop prosthetic-joint tracking.

Surface EMG signals were obtained from one healthy volunteer without lower-limb amputation; the volunteer was not a co-author. The study protocol was approved by the Ethics Committee of Satbayev University (approval No. 10, dated 25 June 2026), and written informed consent was obtained from the volunteer prior to surface EMG data collection. Two Muscle Sensor V3.0 modules (Advancer Technologies, Raleigh, NC, USA) were connected to analog inputs A0 and A1 of the Arduino Uno (Arduino S.r.l., Monza, Italy). Channel 1 was positioned over the rectus femoris region, whereas channel 2 was positioned over an anterior-thigh vastus region. The recordings were used only to demonstrate the sensor-to-controller-to-actuator pathway and did not constitute an evaluation of locomotion-intent recognition, prosthetic walking, or clinical performance.

Both recording sites were within the quadriceps/anterior-thigh region. Their assignment to opposite knee commands was therefore an application-defined input mapping, not a physiologically validated mapping of knee-flexor and knee-extensor activation. In particular, activation recorded over the rectus femoris region does not by itself identify knee-flexion intention. Independent recruitment, cross-talk, fatigue effects, and electrode-placement sensitivity were not quantified. A physiological flexion–extension interface would require an appropriately selected agonist–antagonist muscle configuration and a separate validation protocol.

An Arduino Uno (Arduino S.r.l., Monza, Italy) served as the central controller [24]. The two Muscle Sensor V3.0 outputs were read at A0 and A1; the knee and ankle MG995 servomotors (TowerPro, Taoyuan, Taiwan) were commanded from D9 and D10, respectively; and the GY-521/MPU6050 (TDK InvenSense, San Jose, CA, USA) communicated over I2C through A4/SDA and A5/SCL at address 0 × 68. The standard Arduino Servo and Wire libraries were used [25,26]. The MPU6050 contains accelerometer and gyroscope channels [27], although only accelerometer-derived inclination was used in the command rule reported here. MG995 servomotors were used to demonstrate bench movement of the joint mechanisms [28].

The control loop sequentially acquired the conditioned EMG outputs and accelerometer data, evaluated the threshold rules, updated the servomotor commands, and included a programmed 20 ms delay between loop iterations. This delay provides only a theoretical upper bound of 50 iterations per second before accounting for sensor reads, computation, communication, and other blocking operations; it is not a measured sampling frequency, processing latency, or actuator response time.

**Figure 5:**
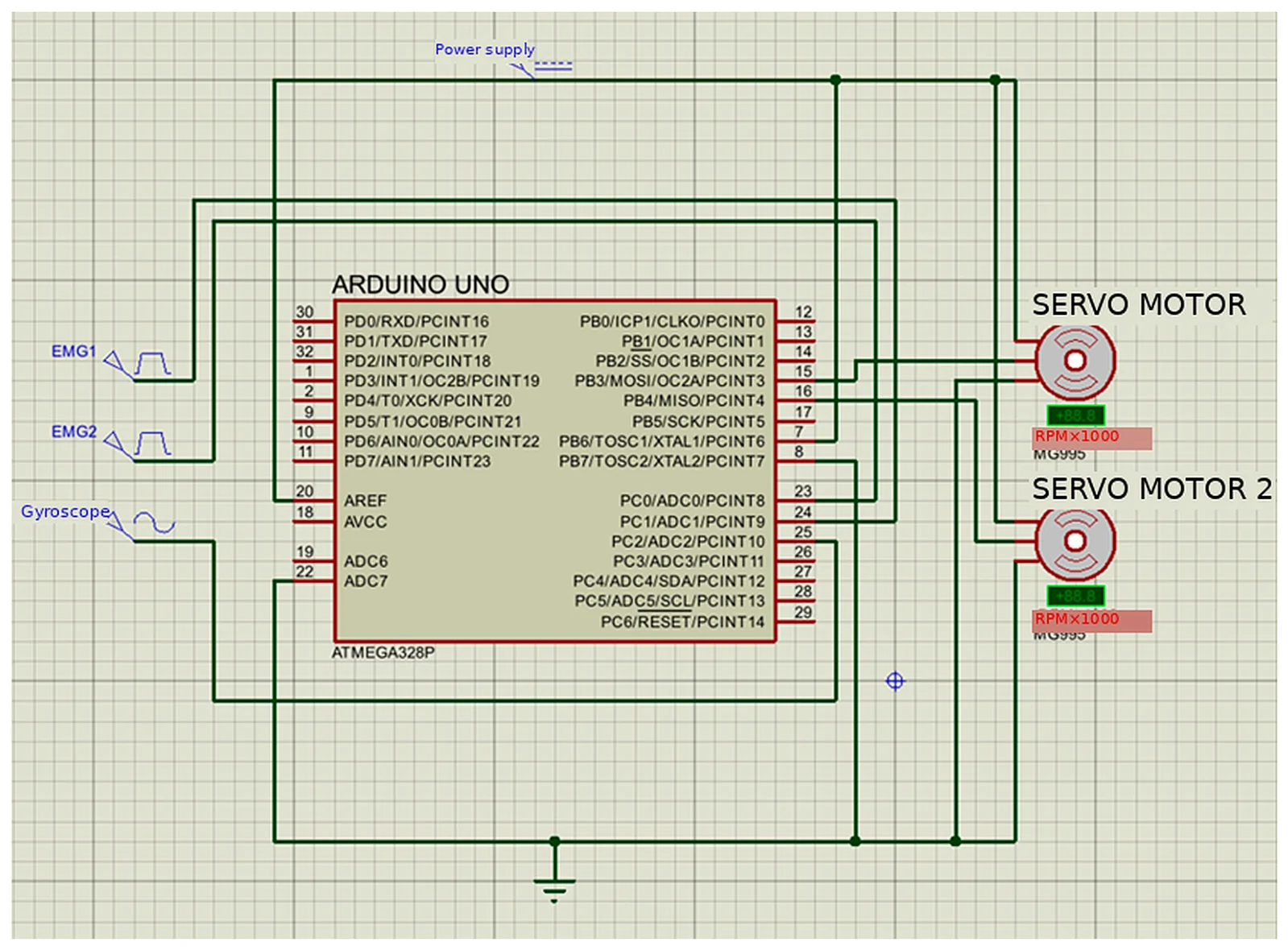
Schematic overview of the electronic architecture. The implemented pin assignments are specified in the text; the figure is not a complete electrical wiring or power-distribution diagram.

The MG995 servomotors were not characterized in the assembled mechanisms for joint torque, mechanical output power, electrical energy consumption, or positioning performance under a specified external load. Manufacturer ratings are therefore not treated as measured actuator output. The demonstrated motion establishes only that the mechanisms could respond to commands on the bench; it does not establish the ability to support a user or to generate physiologically relevant powered knee or ankle motion. The implemented electronic architecture is shown in Figure 5.

The EMG pathway used fixed-threshold switching of the conditioned sensor outputs. An activation threshold of 400 ADC counts was used as an empirical implementation setting. It was not established through a formal calibration or optimization procedure and should not be interpreted as a user-independent physiological activation threshold. The standard Muscle Sensor V3 signal path provides a conditioned output rather than an unprocessed raw surface EMG waveform [29].

The knee–servomotor command was selected from the four possible EMG input states summarized in Table 5. The implemented decision sequence gives channel 1 priority if both inputs exceed the threshold; simultaneous activation was not subjected to a dedicated quantitative response test. This priority is therefore a software decision rule rather than recognition of physiological co-contraction.

$$\begin{array}{c} u_{k}=135{}^{\circ}\;if\;EMG1>400;\\ u_{k}=45{}^{\circ}\;if\;EMG1\leq 400\;and\;EMG2>400;\\ u_{k}=90{}^{\circ}\;if\;EMG1\leq 400\;and\;EMG2\;\leq \;400. \end{array}$$

When both EMG channels exceed the threshold, channel 1 has priority and the 135° knee-servomotor command is selected.

For the ankle pathway, the implemented rule used only the accelerometer channels of the MPU6050. The quantity alpha was calculated from the gravity-sensitive acceleration components in the sensor x–z plane. Under sufficiently slow motion and the assumed sensor alignment, this provides a sensor-frame inclination estimate; it is not an independent measurement of the mechanical ankle angle between the foot and shank.

The inclination estimate was calculated from the accelerometer data as follows:

$$\alpha =arctan2(a_{x}{,a}_{z})\cdot 180/\pi ,$$

where alpha is the sensor-frame inclination estimate and $a_{x}$ and $a_{z}$ are the measured acceleration components along the MPU6050 x- and z-axes, respectively. Translational acceleration, sensor misalignment, and motion outside the assumed plane can change this estimate without an equivalent change in the mechanical ankle angle.

**Figure 6:**
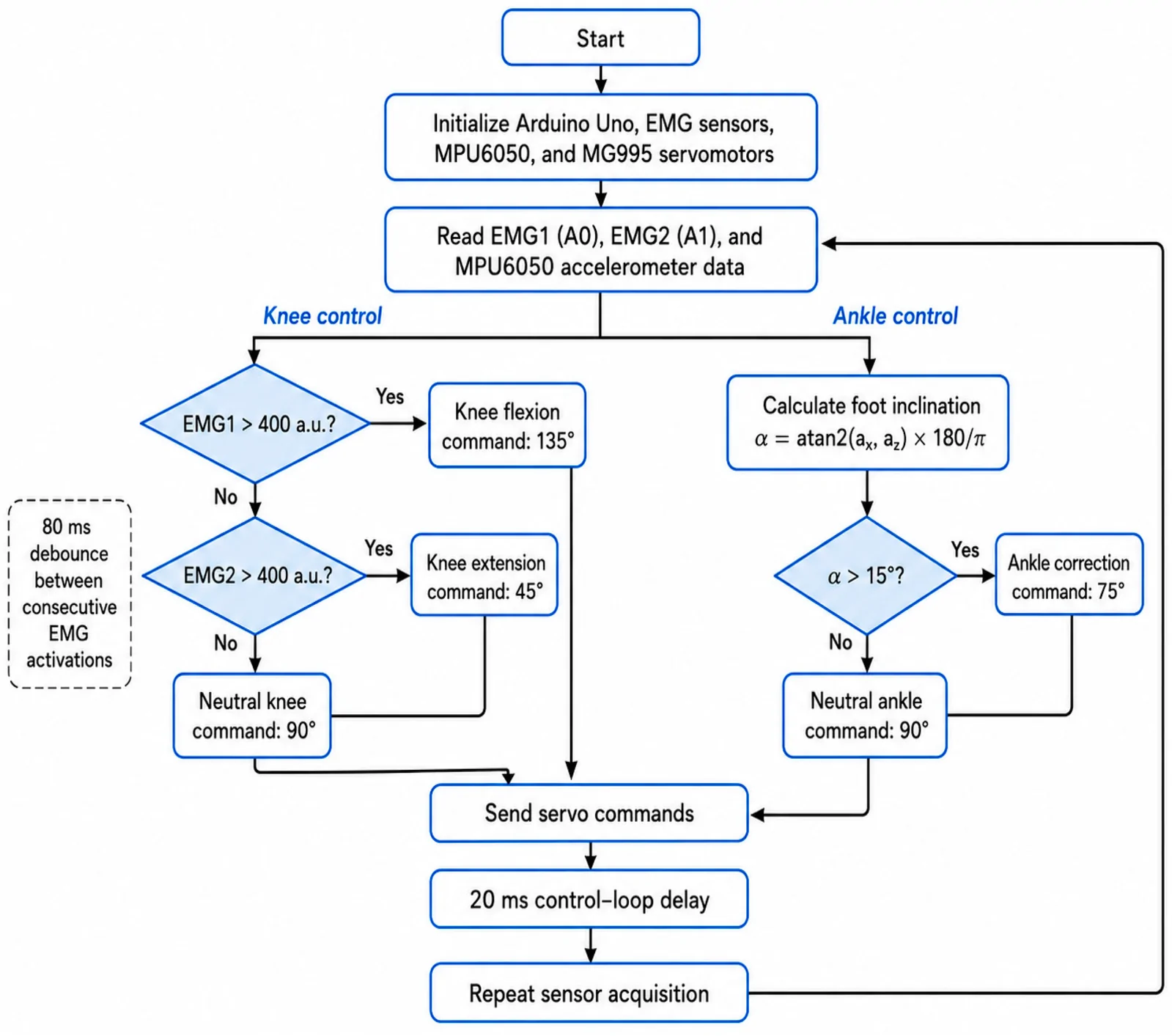
Sensor-triggered command-selection sequence for the prosthetic-leg bench prototype.

The ankle servomotor command was selected according to the following threshold rule:

$$u_{a}=75{}^{\circ}\;for\;\alpha >15{}^{\circ};u_{a}=90{}^{\circ}\;for\;\alpha \leq 15{}^{\circ}.$$

where the stated values are numerical servomotor commands rather than measured ankle joint angles. A 75-degree command was selected when alpha exceeded 15 degrees; otherwise, a 90-degree command was selected. Implementation uses the signed value of alpha and does not establish dynamic ankle stabilization. Gyroscope–accelerometer fusion and comparison with an independent orientation reference are required for dynamic validation. The overall sensor-triggered command-selection sequence is shown in Figure 6.

An 80 ms debounce interval was used as an empirical implementation setting to limit rapid repeated switching. Its effect on missed commands, unintended activations, and overall latency was not quantified. The 400-count threshold, the 80 ms debounce interval, and the 20 ms loop delay are therefore software parameters rather than experimentally optimized performance metrics. The generalized command-selection logic is summarized in Table 5.

Bench evaluation was performed in stages: verification of the electronic signal pathways; qualitative demonstration of EMG-triggered knee commands; qualitative demonstration of inclination-triggered ankle commands; exploratory finite-element studies of selected structural components; separate static compression tests; and an integrated software demonstration in which both input pathways were processed in the same control loop. These stages do not constitute validation of walking, gait coordination, prosthetic-joint tracking, or powered movement under representative user loading.

The CAD models and static finite-element studies were prepared in SOLIDWORKS 2025 (Dassault Systemes, Velizy-Villacoublay, France) using SOLIDWORKS Simulation. Three analyses were reported: (i) a foot model under a 400 N vertical load; (ii) a foot–ankle connection model under 1000 N; and (iii) a load-bearing shank model. Equivalent von Mises stress and resultant displacement were used as engineering response quantities. For PAHT-CF regions, an elastic modulus of approximately 4.0 GPa, a density of 1090 kg/m^3^, and a strength value of 60 MPa were used as modeling assumptions. The 60 MPa value was not established as a specimen-specific allowable for the printed components. Because a complete specimen-specific anisotropic material characterization and a documented mesh-convergence study were not available for the original models, the calculations are interpreted as exploratory estimates for identifying deformation patterns and critical regions rather than as validated predictions of ultimate strength or fatigue life.

The reported numerical and physical tests used different fixtures and loading configurations. Therefore, no quantitative model-test agreement is claimed. A valid future comparison requires reproducing the physical test fixture, support conditions, contacts, load direction, loading range, and displacement measurement location in the numerical model and verifying mesh sensitivity using predefined convergence criteria.

For the present revision, the numerical displacement and the separate compression-test displacement are reported independently. Their ratio is not used as a validation error or as a common stiffness measure because the corresponding boundary conditions and measured quantities differ.

Static compression tests were performed using a WDW-10KN microcomputer-controlled electronic universal testing machine (Jinan Hensgrand Instrument Co., Ltd., Jinan, China) with a nominal capacity of 10 kN. Force and machine displacement were recorded during the reported loading sequences. The flexible-foot specimen was loaded to approximately 1000 N, and the shank–knee assembly reached approximately 1.8 kN in axial compression. The retained dataset contains one reported loading trace for each configuration; consequently, specimen-to-specimen variability, repeated-cycle behavior, and statistical repeatability are not reported. A specimen-linked loading-rate record was not available in the retained dataset. These laboratory loads characterize only the tested configurations and cannot be converted directly into an allowable user body mass or a claim of standards compliance. The complete bench-evaluation program is summarized in Table 6.

The methodology therefore distinguishes between exploratory structural analysis, separate component testing, and qualitative command-pathway demonstrations. The evidence from one stage is not used to infer unmeasured performance at another stage. In particular, no claim is made regarding cyclic durability, clinical safety, population-level EMG performance, joint-angle tracking accuracy, or response under representative gait loading.

Generative AI tools were used for academic language assistance and for the preparation of non-data illustrative graphical materials. They were not used to generate, modify, or analyze experimental measurements, sensor data, or finite-element simulation results. All technical content, numerical values, experimental plots, and interpretations were verified by the authors.

## 3. Results

### 3.1. Results of Numerical Simulation and Mechanical Testing

The numerical studies and physical compression tests are reported as separate exploratory assessments of selected components. They characterize responses under the specified configurations and identify potential design limitations. Because the model and test configurations were not consistently matched, the results do not establish quantitative model agreement or the limiting load capacity of the complete prosthetic-leg prototype.

**Figure 7:**
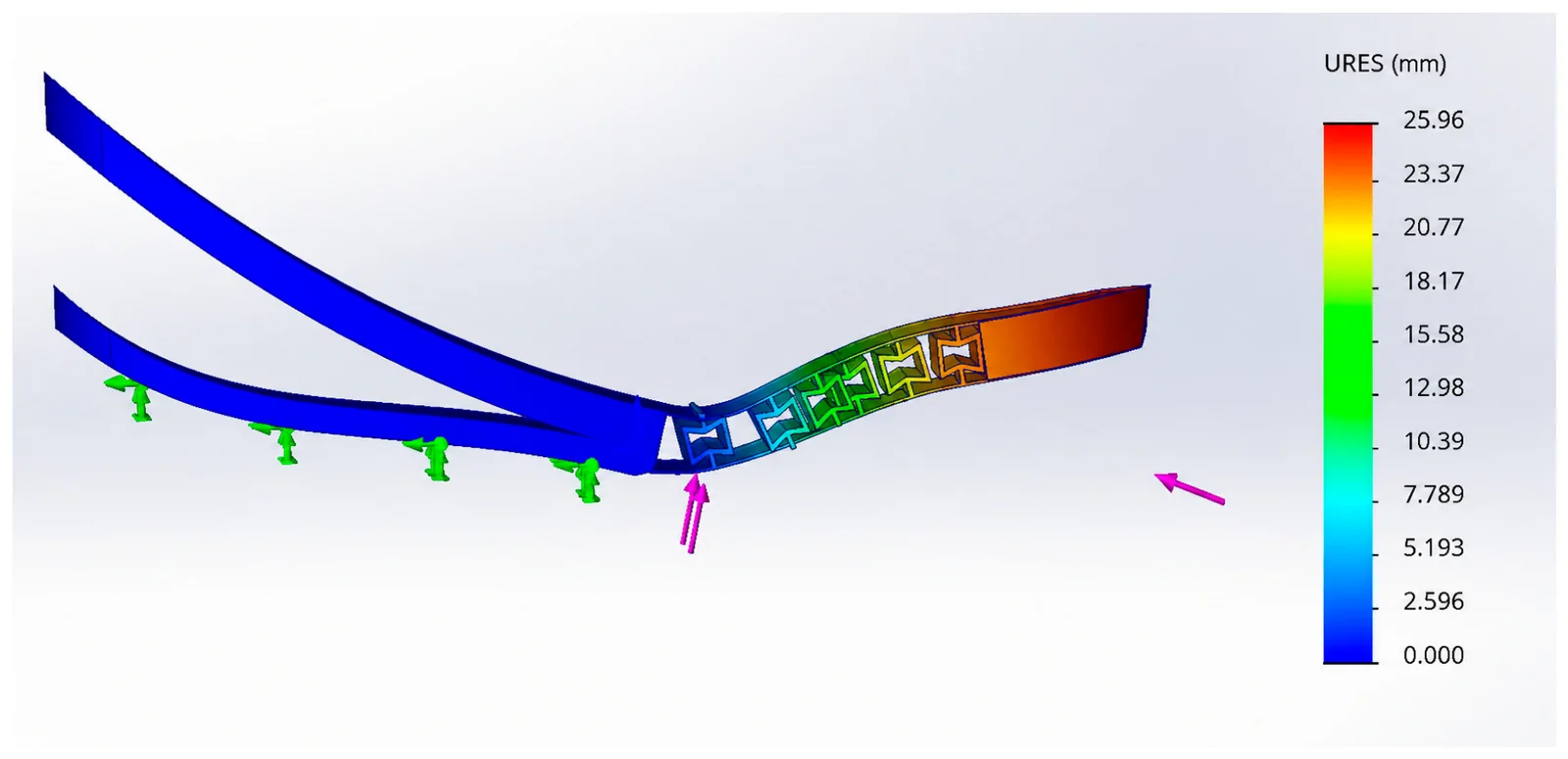
Results of the numerical simulation of the prosthetic foot module under a vertical load of 400 N. Magenta arrows indicate the applied load direction, and green symbols indicate the constrained/support regions.

As described above, numerical simulation of the prosthetic sole was performed under a vertical load of 400 N (Figure 7). The distribution of resultant displacement showed that the majority of deformation was localized within the flexible forefoot segment. The maximum displacement at the free end reached 25.96 mm, whereas the deformation within the functional region of the forefoot segment was approximately 10 mm. In contrast, the heel and midfoot regions exhibited comparatively small displacements, indicating that the load-bearing portion of the structure retained its geometric stability under the applied loading condition.

The displacement field in the foot model is consistent with the intended separation between a relatively rigid structural region and a compliant forefoot region. However, the analysis does not quantify physiological rollover, energy return, impact attenuation, or durability of the printed interface.

**Figure 8:**
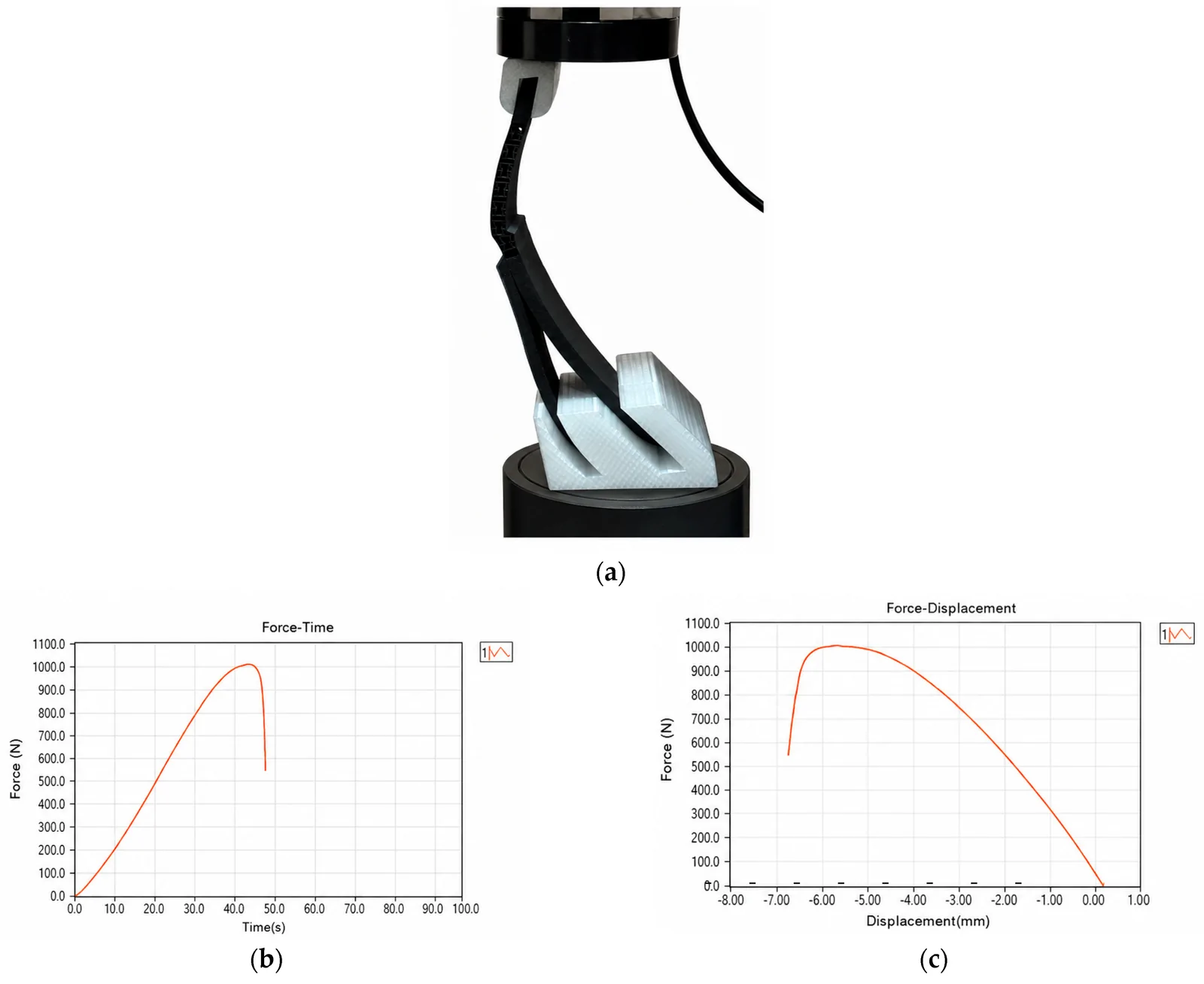
Static testing of the flexible sole: (**a**) specimen positioning in the testing machine; (**b**) force–time relationship; (**c**) force–displacement relationship.

A separate static compression test of the fabricated foot specimen was increased to approximately 1000 N and recorded a maximum machine displacement of approximately 7 mm. No visible cracks or abrupt structural failure were reported during this short-term test. This observation applies only to the specific fixture and loading sequence used. The test configuration and the recorded force histories are shown in Figure 8.

The foot model produced a maximum resultant displacement of 25.96 mm under 400 N, whereas the separate compression test recorded approximately 7 mm at 1000 N. These values describe different loading and support conditions and different displacement measures. Their difference cannot be interpreted as a numerical-model error, and the experimental curve does not quantitatively validate the reported FEA result. Meaningful validation requires a model that reproduces the physical fixture, contacts, load direction, measurement location, and loading range.

**Figure 9:**
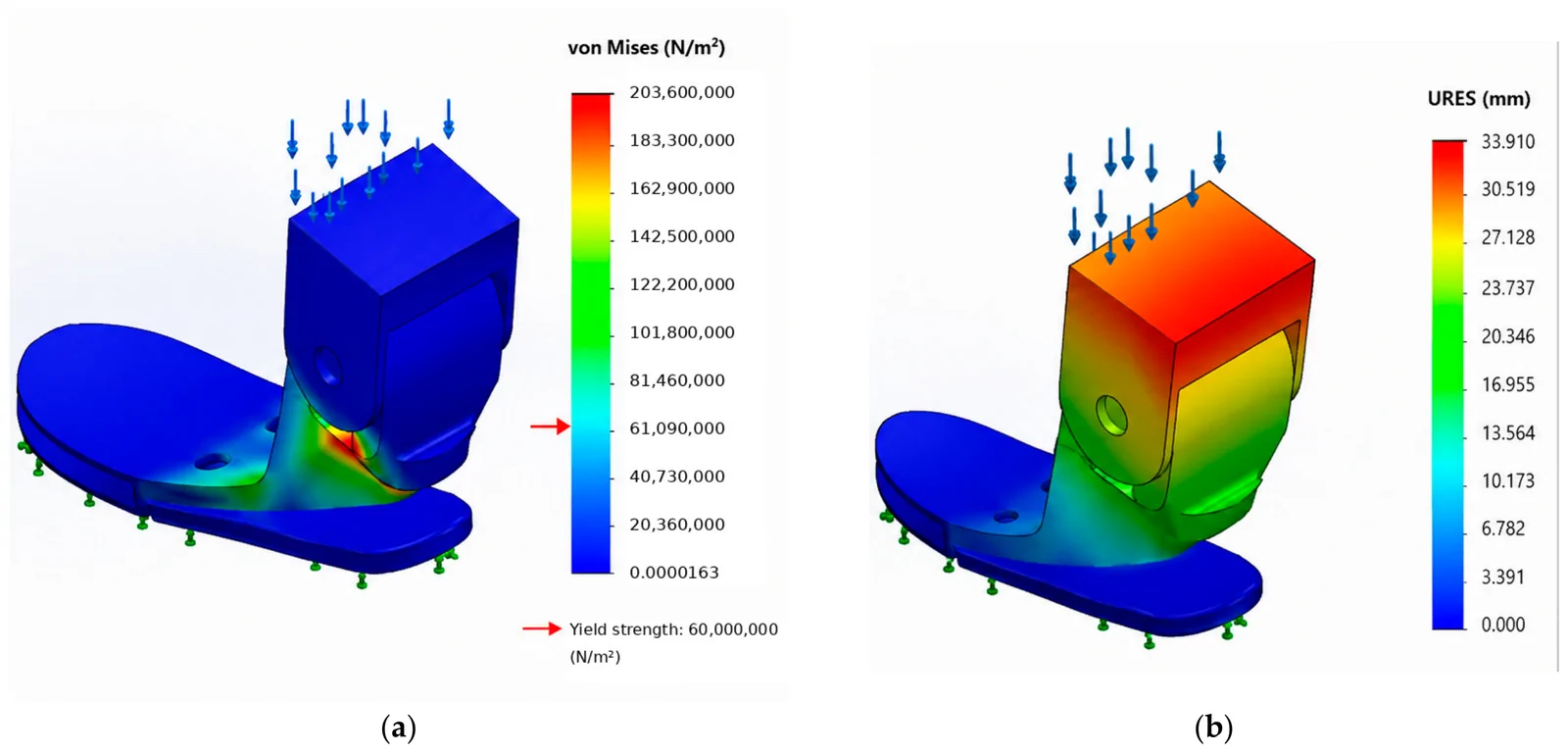
Results of the finite-element analysis of the foot–ankle assembly under a load of 1000 N: (**a**) distribution of equivalent von Mises stress; (**b**) distribution of resultant displacement. Blue arrows indicate the applied load, green symbols indicate constrained regions, and the red arrows in panel (**a**) identify the yield-strength reference on the stress scale.

For the foot–ankle assembly, the equivalent von Mises stress and resultant displacement distributions were determined. Under a simulated load of 1000 N, the maximum resultant displacement reached 33.91 mm. The largest displacement was observed in the upper region of the connecting element, whereas the sole remained nearly stationary in the constrained region. The corresponding stress and displacement fields are shown in Figure 9.

For the foot–ankle connection, the reported maximum equivalent stress was 203.6 MPa under a simulated 1000 N load. Relative to the assumed material-strength value of 60 MPa, the nominal strength-to-stress ratio is 60/203.6, coming to approximately 0.29. This result identifies the connection as a critical limitation of the current geometry under the modeled conditions; it is not an acceptable structural margin and is not used to estimate an allowable user load.

A linear–elastic stress estimate above the assumed material strength cannot be interpreted as a quantitative prediction of the actual post-yield stress state or failure load. The reported displacement of 33.91 mm likewise requires assessment of geometric nonlinearity and model validity. Mesh sensitivity and contact assessment are necessary to distinguish a localized numerical peak from a broader structural deficiency, but they cannot be assumed to remove the problem. The connection requires redesign, reanalysis under documented conditions, and direct mechanical testing before load-bearing performance of the assembled structure can be claimed.

**Figure 10:**
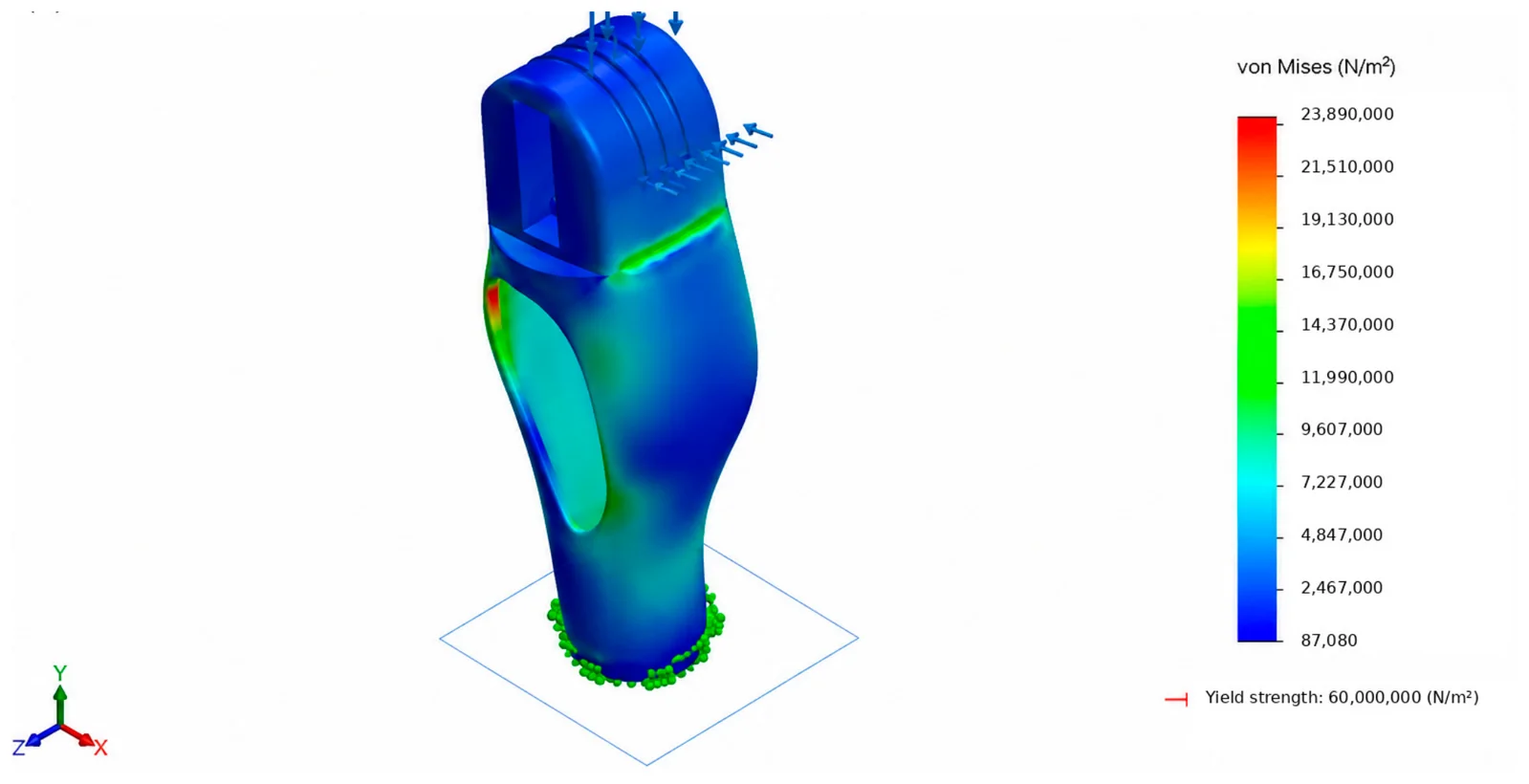
Distribution of equivalent von Mises stresses in the shank structure. Blue arrows indicate the applied load, green symbols indicate constrained regions, and the red arrow identifies the yield-strength reference on the stress scale.

The shank model produced a maximum equivalent stress of 23.89 MPa. Relative to the same assumed 60 MPa material-strength value, the nominal strength-to-stress ratio is approximately 2.51 for this specific model and loading case. This ratio is an engineering indicator based on the stated assumptions; it is not a measured safety factor for the printed shank, the knee connection, or the complete prototype. The shank stress distribution is shown in Figure 10.

**Figure 11:**
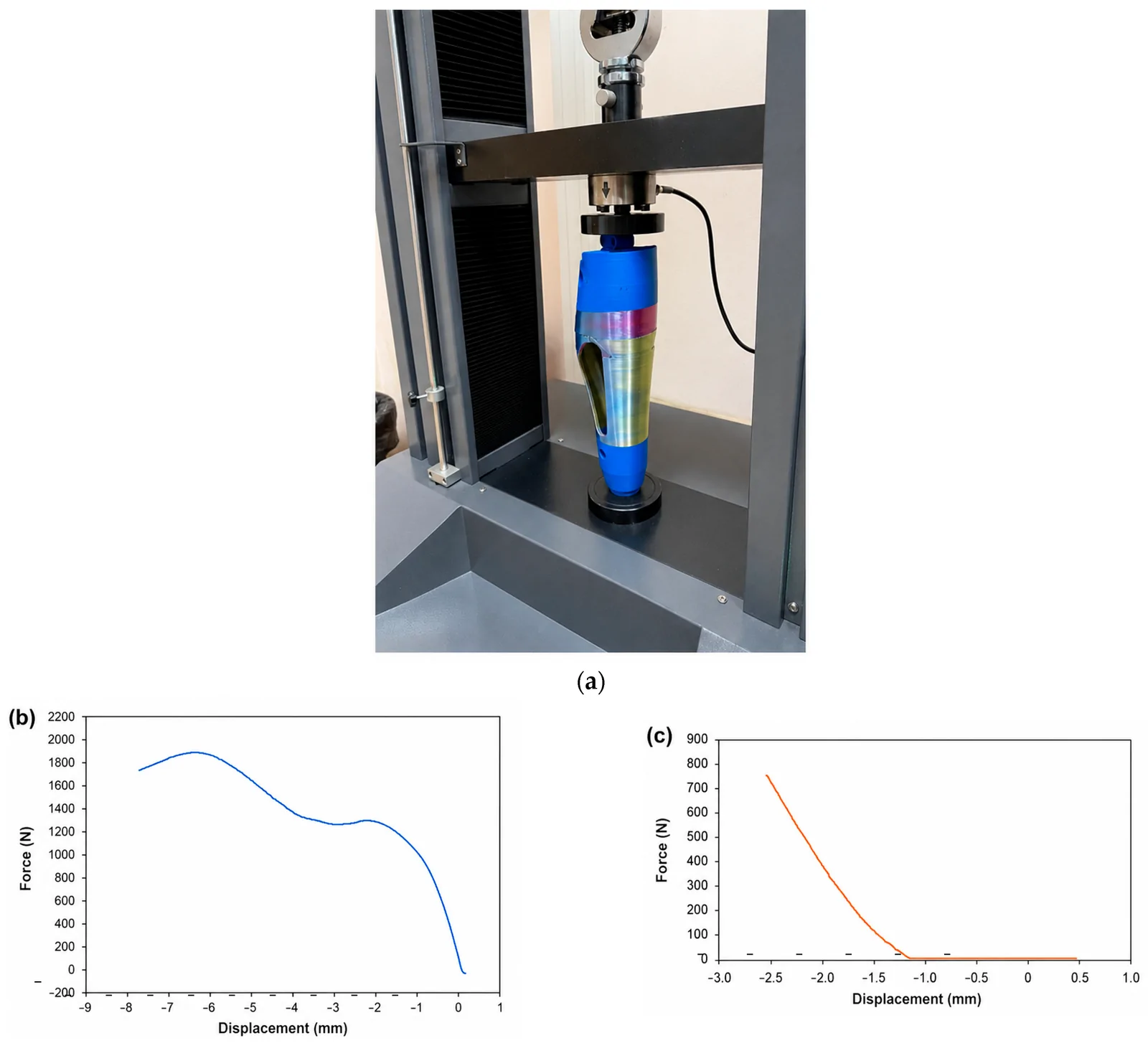
Experimental validation of the shank and knee connection: (**a**) axial compression test configuration; (**b**) force–displacement response of the complete shank–knee assembly; (**c**) force–displacement response of the local knee-connection element.

The shank–knee assembly was evaluated separately under axial compression on the universal testing machine. The specimen was positioned between loading plates so that the applied force acted approximately along its longitudinal axis. This configuration does not reproduce combined bending, torsion, joint moments, or repeated gait loading. The compression configuration and force–displacement curves are shown in Figure 11.

The reported compression curve for the shank–knee assembly reached approximately 1.85 kN at a displacement of approximately 6–7 mm. A separate local knee-connection test reached approximately 0.75 kN at approximately 2.3 mm. These observations belong to different configurations and should not be combined into a single structural performance measure. The absence of an abrupt load drop in the reported traces does not exclude local damage, plastic deformation, or reduced residual strength.

The available observations describe short-term compression behavior only. They do not quantify specimen-to-specimen variability, fatigue life, recovery after unloading, or performance under combined bending and torsion. The structural testing of lower-limb prostheses and ankle–foot devices is addressed by ISO 10328:2016 and ISO 22675:2024 [30,31]; the present experiments were not conducted as conformity tests following those standards.

A summary of the numerical estimates and separate static test observations is presented in Table 7.

The mechanical investigation therefore provides preliminary information on selected components and identifies the foot–ankle connection as a critical unresolved part of the load path. Neither the separate compression tests nor the reported finite-element results establish mechanical validation of the complete assembly. The current geometry is retained in this paper as the evaluated baseline configuration; redesign and re-validation are treated as the next design iteration rather than as evidence already available in the present dataset.

### 3.2. Bench Evaluation of Sensor-Triggered Commands and Integrated Operation

Functional bench demonstrations examined whether the EMG and MPU6050 input pathways generated the programmed servomotor commands. The EMG threshold was 400 ADC counts and the programmed knee–servomotor commands were 45, 90, and 135 degrees. These demonstrations did not include an independent joint-angle reference, a calibrated external load, or synchronized actuator-response instrumentation.

The EMG input pathway produced changes in the knee–servomotor command according to the decision logic in Table 5. The number of repeated transitions, frequency of missed or unintended commands, and measured mechanical response were not quantified. Consequently, the observations demonstrate qualitative command execution rather than validated recognition of natural knee-flexion or knee-extension intention.

**Figure 12:**
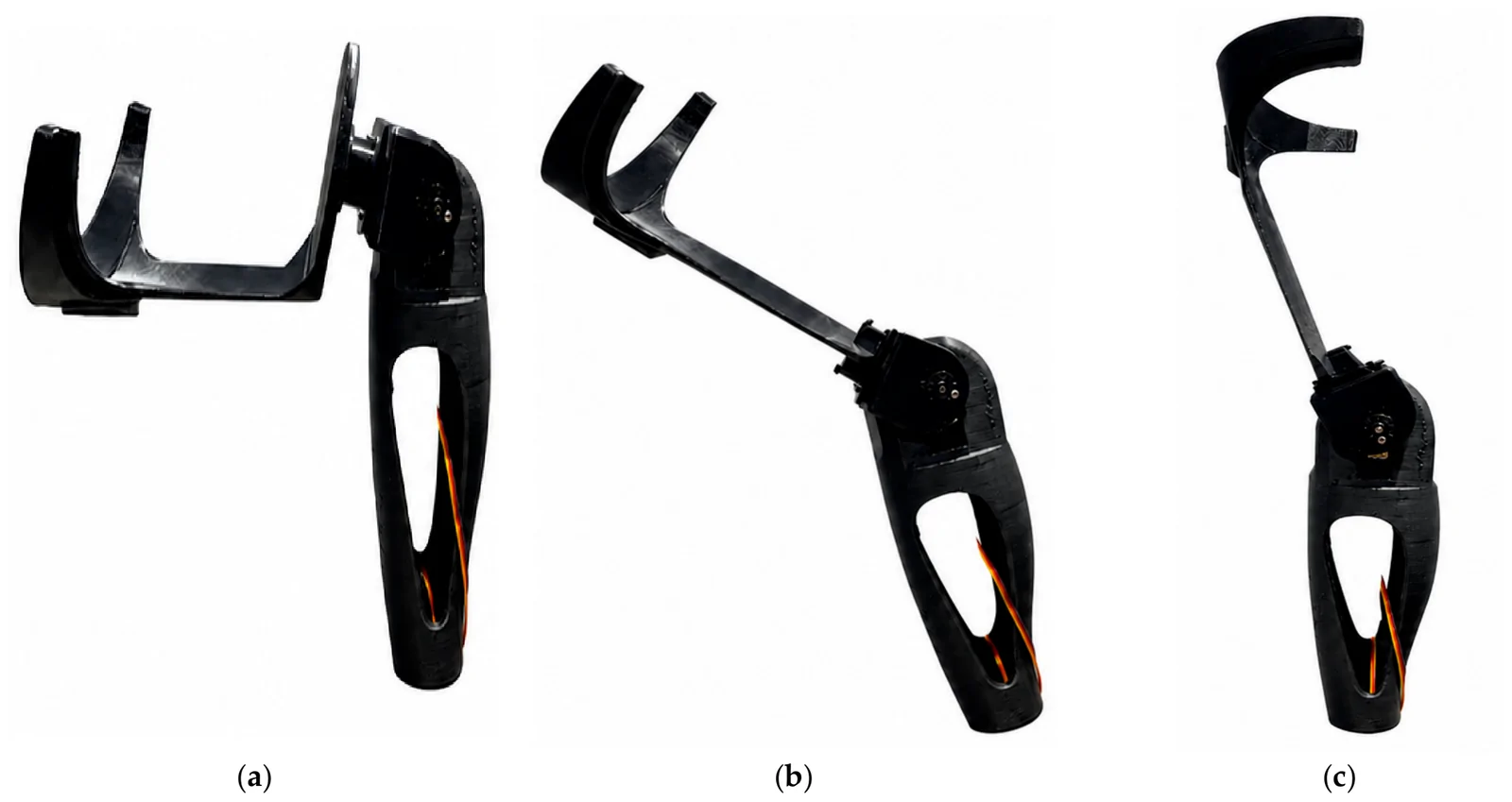
Bench configurations associated with knee–servomotor commands: (**a**) 90 degrees; (**b**) 135 degrees; (**c**) 45 degrees. The stated values are numerical commands and are not independently measured prosthetic-joint angles.

The photographs associated with the three commanded states illustrate configurations reached during the bench demonstration. The stated 45-, 90-, and 135-degree values are numerical servomotor commands and are not independent measurements of the mechanical knee angle. The three commanded bench configurations are shown in Figure 12.

The observed switching confirms continuity of the EMG-to-controller-to-servomotor command pathway under the reported bench conditions. It does not establish positioning accuracy, command repeatability, user-independent threshold performance, or gait-control capability.

The 400-count threshold and 80 ms debounce interval were empirical implementation settings. No receiver-operating analysis, repeated-trial success rate, false-trigger rate, or electrode-repositioning study was available. Quantitative evaluation of these quantities is required before the EMG pathway can be characterized as reliable or repeatable.

**Figure 13:**
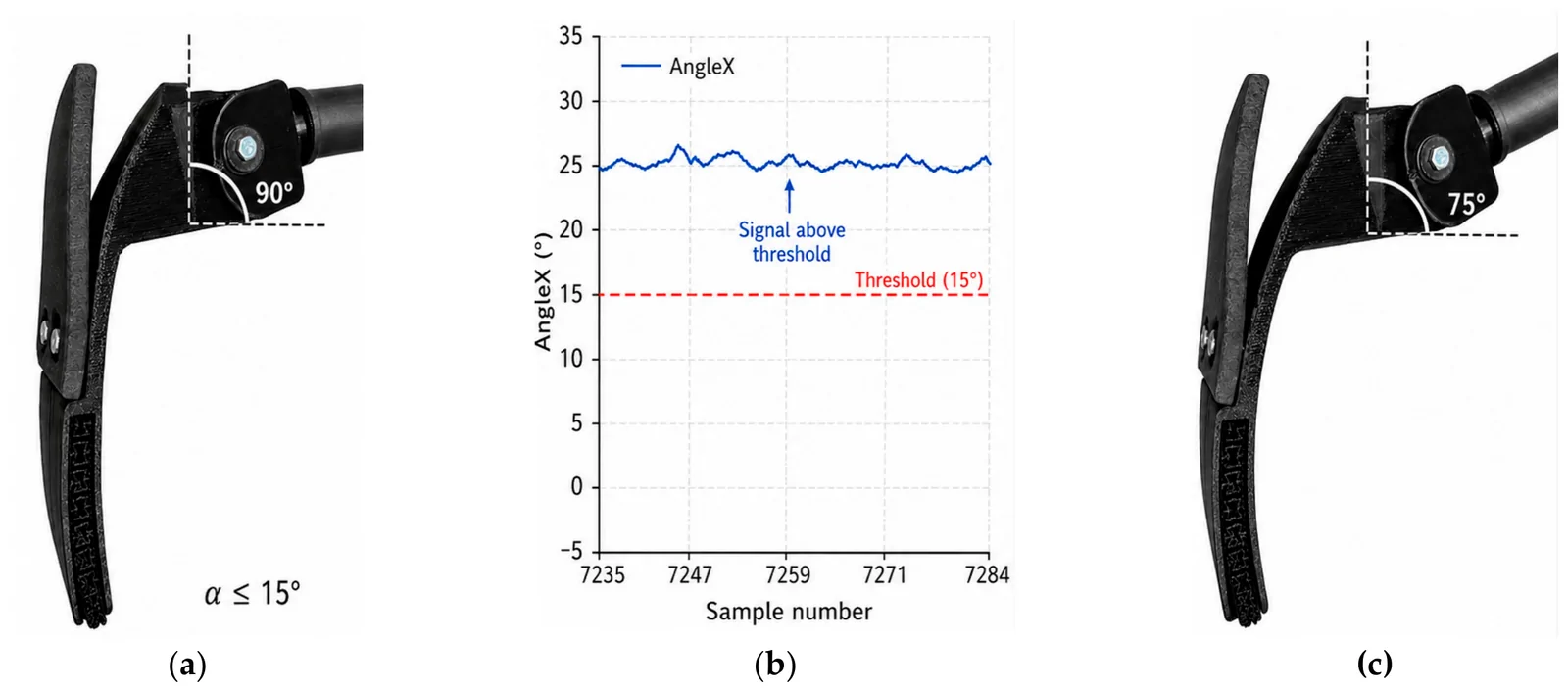
Bench demonstration of inclination-triggered ankle commands: (**a**) alpha <= 15 degrees with a 90-degree servomotor command; (**b**) alpha above the 15-degree threshold; (**c**) a 75-degree servomotor command. Command values are numerical servomotor commands rather than independently measured ankle angles.

The ankle pathway was demonstrated by changing the orientation of the MPU6050 and applying the stated inclination threshold. The controller selected a 75-degree servomotor command when alpha exceeded 15 degrees and a 90-degree command otherwise. The experiment did not independently measure the mechanical ankle angle, establish the accuracy of alpha, or evaluate orientation estimation during dynamic motion. The inclination-triggered demonstration is shown in Figure 13.

Accordingly, the observed switching demonstrates operation of the sensor-input-to-command pathway rather than validated ankle stabilization. Because the estimator uses accelerometer data only, dynamic acceleration can alter the calculated inclination. Gyroscope–accelerometer fusion and an independent orientation reference are required for quantitative dynamic evaluation.

A single sensor-frame inclination also does not establish the relative foot–shank angle. Direct prosthetic-joint tracking would require either a joint-angle sensor or suitably calibrated relative orientation measurements of the foot and shank.

During the integrated demonstration, the Arduino sequentially acquired both EMG inputs and the accelerometer signal and generated commands for both servomotors within the same software loop. The programmed 20 ms loop delay and 80 ms debounce interval are implementation parameters; they cannot substitute for measured onset latency, settling time, steady-state error, or control bandwidth.

**Figure 14:**
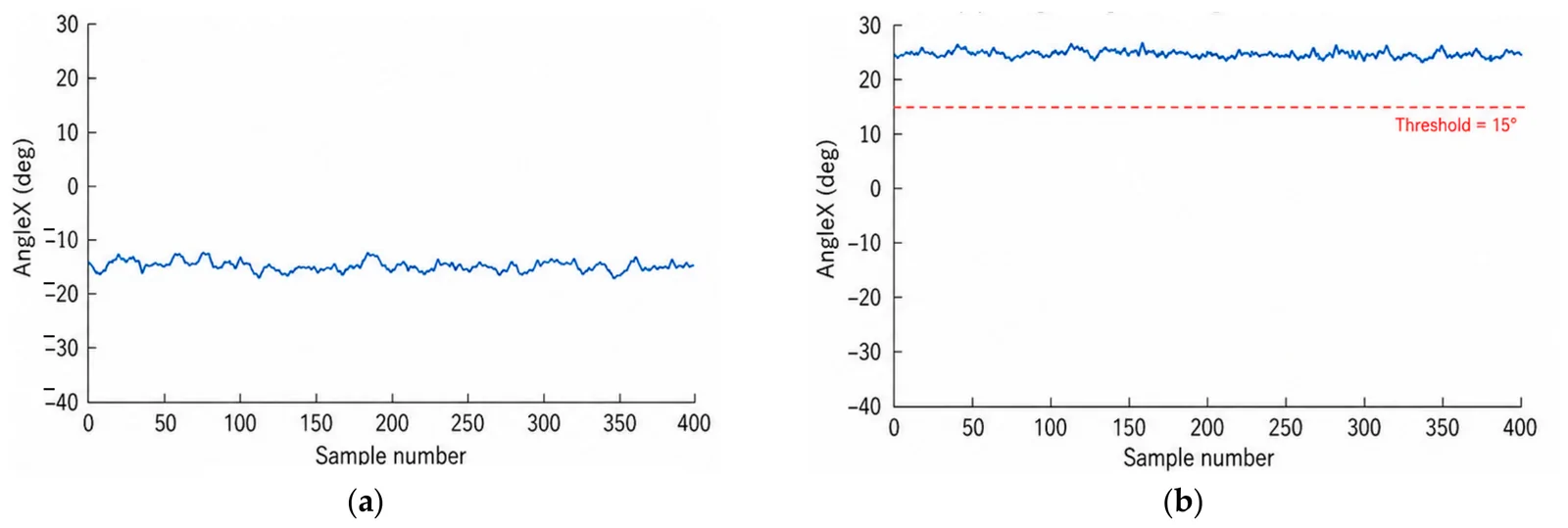

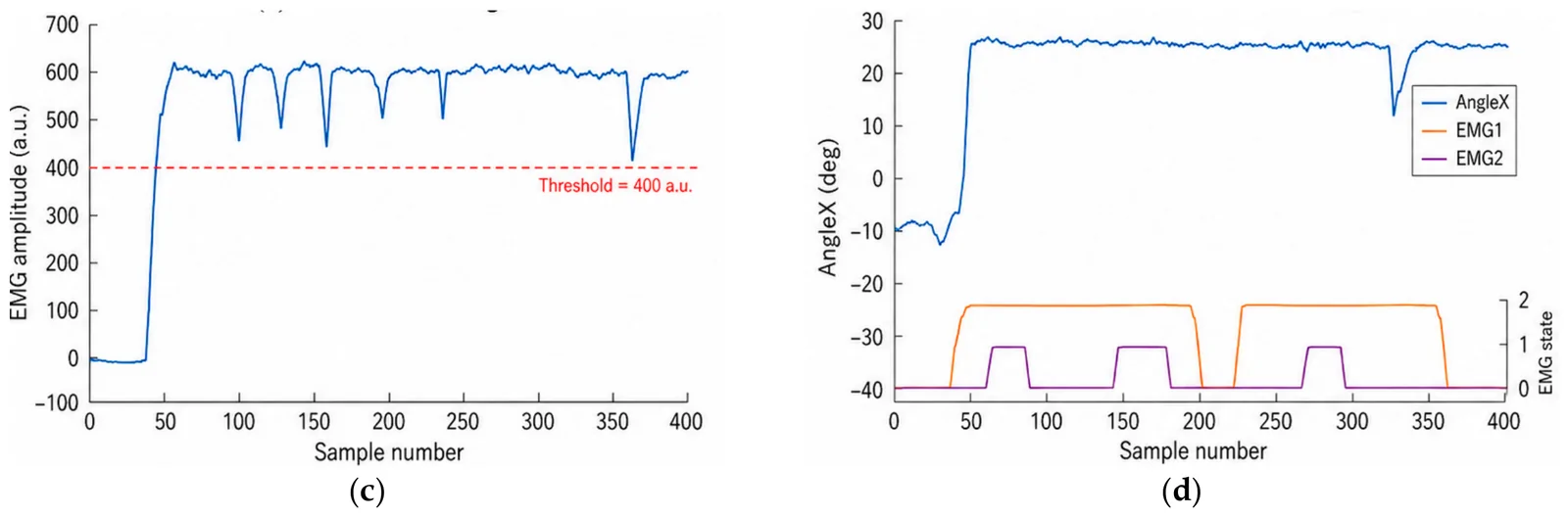
Representative controller inputs: (**a**) AngleX below the positive threshold; (**b**) AngleX above 15 degrees; (**c**) conditioned EMG output relative to 400 ADC counts; (**d**) AngleX and encoded EMG input states. The horizontal axis is sample number; the plotted records do not include independent joint-position measurements.

The integrated test therefore demonstrates the software coexistence of the two command pathways and qualitative actuator response. It does not establish gait coordination, mechanical tracking accuracy, powered movement under representative load, or synchronized multi-joint control performance (Figure 14).

The qualitative bench observations are summarized in Table 8.

No quantitative joint tracking, electrical power, controlled-load actuator performance, or synchronized response-time measurements were available for the reported functional demonstrations. The current observations therefore support only preliminary sensor-triggered operation of the laboratory prototype.

The functional results should be interpreted as an engineering feasibility demonstration. Repeated command trials on one participant would characterize only within-participant switching repeatability and would not establish population-level performance. Testing with individuals with lower-limb amputation, gait analysis, or human load-bearing use should be considered only after the mechanical load path and quantitative controller performance have been established.

## 4. Discussion

The present study examined a prosthetic-leg bench prototype integrating a multi-material foot with separately commanded knee and ankle mechanisms. Its contribution lies in documenting this configuration and the limitations encountered during preliminary mechanical and functional evaluation. EMG sensing, inertial measurements, servomotor actuation, and powered prosthetic joints are established technologies; the results therefore do not establish a new sensing principle, a new physiological intent-recognition method, or improved control performance over mature, powered platforms [8,20]. Relative to our previous knee-centered study [9], the present work is primarily a hardware-scope extension to the foot–ankle–knee chain.

The multi-material foot separates a relatively rigid structural region from a compliant forefoot region, which is consistent with the broader interest in geometrically tailored compliance in additively manufactured prosthetic feet [7]. However, the present study does not quantify energy return, impact attenuation, rollover mechanics, or manufacturing repeatability. The numerical foot displacement and the separate compression-test displacement were obtained under different boundary conditions and cannot be used to judge model accuracy. This distinction is important because a design feature that produces compliance in one fixture does not, by itself, demonstrate physiological foot function.

The foot–ankle connection is the principal unresolved structural limitation. Its reported stress estimate of 203.6 MPa exceeds the assumed 60 MPa strength value, and no direct physical test of this connection was included. The nominal ratio below one identifies a design problem rather than a validated failure load. Although the reviewer-recommended redesign is technically appropriate, it would constitute a new design iteration requiring new geometry, manufacturing, finite-element analysis, and mechanical testing. The current paper therefore retains the tested baseline geometry, explicitly reports its failure to satisfy the adopted modeling criterion, and removes any claim that the complete assembly has been mechanically validated.

The shank model and the separate axial-compression test provide useful preliminary information, but they do not compensate for the unresolved foot–ankle load path. The reported short-term axial response does not reproduce the repeated compression, bending, torsion, and joint moments encountered in prosthetic use. Future structural work should first redesign and directly test the foot–ankle connection, and then perform matched model-test comparisons, multiple-specimen testing, and repeated loading under documented force and moment combinations. ISO 10328:2016 and ISO 22675:2024 provide relevant frameworks for structural and ankle–foot testing [30,31], but the present experiments should not be interpreted as standards-conformity tests.

The EMG experiment demonstrates an application-defined mapping from two anterior-thigh recording sites to discrete servomotor commands. Because both sites were in the quadriceps/anterior-thigh region, the channels cannot be interpreted as a validated knee-flexor/knee-extensor interface. Fixed-threshold switching can be affected by electrode placement, cross-talk, fatigue, signal gain, and inter-individual variation. A stronger next-stage protocol should define anatomical placement, perform participant-specific calibration, quantify correct, missed, and unintended commands across repeated trials, and evaluate robustness after electrode repositioning. Such experiments are necessary before the system can be described as recognizing physiological movement intention.

The accelerometer-derived alpha variable provides a restricted sensor-frame inclination estimate and was used only to switch between two ankle–servomotor commands. Dynamic acceleration can alter this estimate, and a single sensor inclination does not establish the relative foot–shank angle. In addition, the controller did not compare a measured prosthetic-joint position with a joint reference. Quantitative controller evaluation should therefore distinguish sensor-estimation error, command-generation latency, mechanical tracking error, and actuator behavior under load. Independent joint-angle sensing, synchronized timestamps, controlled external moments, electrical power measurement, and comparison of accelerometer-only versus fused orientation estimates are appropriate technical milestones.

The transition from this laboratory platform to a clinically relevant prosthetic system requires a staged validation pathway: (1) redesign and direct testing of the foot–ankle connection; (2) matched FEA-experiment comparison with controlled manufacturing records; (3) repeated and cyclic structural testing; (4) independent measurement of knee and ankle joint motion, actuator torque, electrical consumption, response time, and positioning error under controlled load; (5) robust EMG/IMU estimation with calibrated sensor fusion and continuous or impedance-based control; and, only then, (6) ethically approved human gait and load-bearing studies. The current evidence supports continued bench research on the architecture but does not support safe human load-bearing use or clinically validated prosthetic function.

## 5. Conclusions

A sensor-integrated prosthetic-leg bench prototype was developed using a PAHT-CF/TPU 95A foot, articulated knee and ankle mechanisms, two EMG inputs, an MPU6050, an Arduino Uno, and MG995 servomotors. The functional demonstrations showed that the sensor inputs could trigger discrete actuator commands within an integrated software implementation. They did not quantify prosthetic-joint tracking, response time, repeatability, actuator output under representative load, or walking performance.

The numerical studies and separate compression tests provided preliminary information on selected structural components. Their different loading configurations prevent quantitative FEA validation. The foot–ankle connection was identified as a critical unresolved component, with a reported stress estimate of 203.6 MPa relative to an assumed strength value of 60 MPa. The separate shank–knee compression result does not establish mechanical reliability of the complete assembly.

The present work therefore supports continued bench investigation of the configuration rather than clinical or load-bearing claims. Structural redesign, matched numerical and experimental evaluation, repeated loading, controlled manufacturing documentation, independent joint-performance measurements, and quantitative sensor/controller testing are required to establish operating limits. No conclusion regarding safe human load-bearing use or gait performance can be drawn from the current results.

## Figures and Tables

**Table 1:** Direct comparison of the study scope with related prosthetic platforms.

Feature	Previous Knee Prototype [9]	Open-Source Bionic Leg [8]	Present Prototype
Main scope	Semi-active knee architecture	Powered knee-and-ankle research platform	Articulated prosthetic-leg bench prototype
Sensing/control evidence	EMG and IMU described	Embedded sensing with quantitative controller characterization	Two EMG inputs and accelerometer-derived inclination used for discrete commands
Foot/ankle scope	Knee-centered investigation	Powered ankle with integrated foot	PAHT-CF/TPU 95A foot and servo-actuated ankle mechanism
Evaluation depth	Previously reported knee-module bench investigation	Electromechanical bench testing and participant ambulation	Separate component compression tests and qualitative actuator demonstrations
Claim supported by current evidence	Starting point for the present hardware extension	Benchmark for a mature powered research platform	Hardware integration extension; no demonstrated performance superiority

**Table 2:** Comparative analysis of contemporary approaches to active lower-limb prosthesis design.

№	Reference	Approach	Main Concept	Advantages	Limitations	Application in the Present Study
1	[10]	Microprocessor-controlled knee modules	Controlled damping of the knee joint	Improved stability and safety	High cost, user-specific tuning, specialized maintenance, and limited positive power generation	The need for adaptive control was considered
2	[11]	Active lower-limb prostheses	The prosthesis is considered as an integrated mechatronic system	Integrated approach to actuation and control	Increased mass, power consumption, cost, safety requirements, and control complexity	The system-level prosthetic architecture was substantiated
3	[12]	Powered knee–ankle prosthesis	Powered knee and ankle joints	Ability to generate active motion at two joints	High actuator power demand, increased system mass, and complex multi-joint coordination	Coordinated operation of the knee and ankle joints was incorporated
4	[13]	Powered knee prosthesis	Controlled knee flexion and extension	Improved gait symmetry	Actuator and transmission complexity and the need for accurate joint-position control	The use of an active knee joint was substantiated
5	[14]	Powered ankle prosthesis	Power generation during push-off	Improved walking energetics	Increased energy consumption and additional actuator and battery mass	The role of the ankle joint during the gait cycle was considered
6	[8]	Open-source bionic leg	Modular powered prosthetic platform	Reproducibility and research value	High technical complexity and the need for precise actuator design and controller tuning	The principle of modular architecture was incorporated
7	[15]	Control strategies	Finite-state machines, impedance control, and phase-variable control	Adaptability to different gait phases	Requires reliable gait-state estimation and user-specific parameter tuning	The need for an appropriate control logic was substantiated
8	[16]	User-intent recognition	Real-time multiclass control	Enables locomotion-mode selection	Classification errors may occur during locomotion transitions; calibration and robust sensor input are required	The relationship between sensor data and user intent was considered
9	[17]	EMG-based control of a powered prosthetic leg	Use of EMG signals and previous gait-cycle information	Improved intuitiveness of control	Sensitivity to EMG variability, electrode placement, noise, and individual calibration	The use of EMG signals was substantiated
10	[18]	EMG and mechanical sensor fusion	fusion of biosignals and mechanical sensor data	Improved recognition accuracy	Increased sensor count, synchronization requirements, and calibration complexity	A multimodal sensor architecture was employed
11	[19]	Sensory feedback	Vibrotactile gait feedback	Improved user–prosthesis interaction	Requires additional feedback hardware and user adaptation; effectiveness depends on feedback tuning	Considered as a direction for future development
12	[20]	Robotic lower-limb prosthesis	Integration of mechanical design, actuation, and control	Holistic engineering approach	High system-integration complexity, mass and energy constraints, and extensive validation requirements	The integration of mechanical and electronic subsystems was substantiated
13	[21]	Phase-based control	Continuous gait-phase-based motion control	Smoother prosthetic operation	Requires reliable estimation of the gait phase and accurate user-specific tuning	Considered as a prospective direction for further algorithm development
14	[22]	Locomotion-mode recognition	Decision delay during locomotion-mode transitions	Improved classification accuracy	Decision delay may improve classification accuracy but can reduce responsiveness during transitions	Used as a reference for future improvements
15	[23]	Robotic knee prosthesis	Optimization of the knee joint mechanism	Improved mechanical transmission efficiency	Mechanical complexity, precision-manufacturing requirements, and transmission optimization	The importance of knee-joint kinematics was substantiated

**Table 3:** Main structural components of the prototype.

Prosthetic Component	Structural Role	Implementation Feature
Prosthetic Socket	Interface between the prosthesis and the user	Upper interface for load transmission
Knee Joint	Flexion and extension of the lower limb	Articulated joint designed to accommodate an actuator
Shank	Load transmission between the knee and foot	Load-bearing connecting element
Ankle Joint	Adjustment of foot position relative to the shank	Revolute connection between the foot module and the shank
Prosthetic Foot Module	Support and intended forefoot compliance	Hybrid structure with a flexible forefoot segment
Electronic Control Unit	Integration of control components	Housing for the microcontroller, sensors, and interconnection elements

**Table 4:** Materials and functional roles of the prosthetic foot module.

Material	Application Area	Key Properties	Functional Purpose
PAHT-CF (Bambu Lab, Shenzhen, China)	Load-bearing section of the foot module and rigid structural components	High stiffness, mechanical strength, and dimensional stability	Load bearing and preservation of structural geometry
TPU 95A (Bambu Lab, Shenzhen, China)	Flexible forefoot segment	Elasticity and reversible deformation	Passive compliance of the forefoot region
PAHT-CF/TPU 95A	Hybrid prosthetic foot module	Combination of rigid and flexible regions	Functional separation of load-bearing and deformable regions

**Table 5:** Sensor-triggered command-selection logic.

Joint	Input Condition	Numerical Servomotor Command	Interpretation
Knee	EMG1 > 400; EMG2 <= 400	135 degrees	Channel 1 selected
Knee	EMG1 <= 400; EMG2 > 400	45 degrees	Channel 2 selected
Knee	EMG1 <= 400; EMG2 <= 400	90 degrees	Default command
Knee	EMG1 > 400; EMG2 > 400	135 degrees	Channel 1 priority; concurrent activation not quantitatively evaluated
Ankle	alpha > 15 degrees	75 degrees	Threshold-triggered command
Ankle	alpha <= 15 degrees	90 degrees	Default command

**Table 6:** Bench evaluation program.

Stage	Test Object	Method	Evidence/Quantity Addressed
1	Electronic circuit	Connection and signal-path verification	Power/signal continuity and command generation
2	EMG pathway	Threshold-triggered servomotor commands	Qualitative switching among 45-, 90-, and 135-degree knee-servo commands; no measured joint angle or latency
3	MPU6050 pathway	Inclination-threshold switching	Accelerometer-derived alpha and 75/90-degree ankle-servo commands; no dynamic orientation validation
4	Foot/foot–ankle/shank models	SOLIDWORKS Simulation	Exploratory displacement/stress estimates under stated loads
5	Fabricated components	WDW-10KN static compression	Separate force–displacement observations; no fatigue or matched FEA validation
6	Integrated controller	Both input pathways processed in one loop	Software integration and qualitative actuator response; no gait or loaded multi-joint validation

**Table 7:** Numerical estimates and separate static test observations.

Structural Component	Numerical Simulation Result	Physical Test Result	Main Conclusion
Flexible sole and forefoot segment	Under a load of 400 N, the maximum displacement of the free end was 25.96 mm, while displacement within the functional region of the flexible segment was approximately 10 mm	Under loading up to 1000 N, the maximum displacement was approximately 7 mm; no structural failure or pronounced residual deformation was observed	Separate numerical and compression-test observations; quantitative FEA validation was not performed.
Foot–ankle assembly	Under a load of 1000 N, the maximum resultant displacement was 33.91 mm; the maximum equivalent stress was 203.6 MPa, compared with an assumed yield strength of 60 MPa	No direct static test of this assembly was available in the presented dataset	Critical modeled stress and displacement; no direct component test; redesign and mechanical evaluation are required.
Shank and knee connection	Maximum equivalent stress 23.89 MPa; nominal strength-to-stress ratio approximately 2.51 relative to the assumed 60 MPa value for the stated model.	The maximum axial compressive force was approximately 1850 N, with a displacement of approximately 6–7 mm	Preliminary model estimate and separate short-term compression response; no verified system safety factor.
Local knee-connection element	A separate numerical analysis for this loading mode was not available in the presented data	The maximum force was approximately 750 N at a displacement of approximately 2.3 mm	Separate local compression observation without a matched numerical model.

**Table 8:** Qualitative bench observations of sensor-triggered commands.

Test Pathway	Programmed Action	Evidence Presented	Not Established
EMG channel 1	Select 135-degree knee–servomotor command	Sensor trace and qualitative movement demonstration	Mechanical joint angle, latency, false-trigger rate
EMG channel 2	Select 45-degree knee–servomotor command subject to decision priority	Qualitative movement demonstration	Mechanical joint angle, latency, missed-command rate
Both EMG inputs ≤ threshold	Select 90-degree knee–servomotor command	Reported default-state behavior	Positioning repeatability
Both EMG inputs > threshold	Apply channel-1-priority rule	No dedicated quantitative response test reported	Response to concurrent activation
Inclination alpha > 15 degrees	Select 75-degree ankle–servomotor command	Inclination trace and qualitative demonstration	Actual ankle angle and dynamic estimation accuracy
Inclination alpha ≤ 15 degrees	Select 90-degree ankle–servomotor command	Reported default-state behavior	Reference-angle accuracy and stability
Integrated controller	Update both servomotor commands in one software loop	Combined input acquisition and bench movement	Quantitative multi-joint performance under load

## Data Availability

The engineering data supporting the reported bench study are available from the corresponding author upon reasonable request. Public sharing of participant-derived EMG recordings is subject to the verified consent terms and applicable institutional requirements.

## Abbreviations

EMG	Electromyography
IMU	Inertial Measurement Unit
PWM	Pulse-Width Modulation
I2C	Inter-Integrated Circuit
CAD	Computer-Aided Design
FEA	Finite Element Analysis
PAHT-CF	High-Temperature Polyamide Reinforced with Carbon Fiber
TPU	Thermoplastic Polyurethane
ADC	Analog-to-Digital Converter
a.u.	Arbitrary Units
WHO	World Health Organization
UNICEF	United Nations Children’s Fund
